# A flexible, interpretable, and accurate approach for imputing the expression of unmeasured genes

**DOI:** 10.1093/nar/gkaa881

**Published:** 2020-10-19

**Authors:** Christopher A Mancuso, Jacob L Canfield, Deepak Singla, Arjun Krishnan

**Affiliations:** Department of Computational Mathematics, Science and Engineering, Michigan State University, East Lansing, MI 48824, USA; Department of Computational Mathematics, Science and Engineering, Michigan State University, East Lansing, MI 48824, USA; Department of Biochemistry and Molecular Biology, Michigan State University, East Lansing, MI 48824, USA; Department of Computational Mathematics, Science and Engineering, Michigan State University, East Lansing, MI 48824, USA; Indian Institute of Technology, Delhi, India; Department of Computational Mathematics, Science and Engineering, Michigan State University, East Lansing, MI 48824, USA; Department of Biochemistry and Molecular Biology, Michigan State University, East Lansing, MI 48824, USA

## Abstract

While there are >2 million publicly-available human microarray gene-expression profiles, these profiles were measured using a variety of platforms that each cover a pre-defined, limited set of genes. Therefore, key to reanalyzing and integrating this massive data collection are methods that can computationally reconstitute the complete transcriptome in partially-measured microarray samples by imputing the expression of unmeasured genes. Current state-of-the-art imputation methods are tailored to samples from a specific platform and rely on gene-gene relationships regardless of the biological context of the target sample. We show that sparse regression models that capture sample-sample relationships (termed *SampleLASSO*), built on-the-fly for each new target sample to be imputed, outperform models based on fixed gene relationships. Extensive evaluation involving three machine learning algorithms (LASSO, k-nearest-neighbors, and deep-neural-networks), two gene subsets (GPL96–570 and LINCS), and multiple imputation tasks (within and across microarray/RNA-seq datasets) establishes that *SampleLASSO* is the most accurate model. Additionally, we demonstrate the biological interpretability of this method by showing that, for imputing a target sample from a certain tissue, *SampleLASSO* automatically leverages training samples from the same tissue. Thus, *SampleLASSO* is a simple, yet powerful and flexible approach for harmonizing large-scale gene-expression data.

## INTRODUCTION

High-throughput gene expression technologies—especially microarray (1) and RNA-sequencing (RNA-seq) (2)—have revolutionized our ability to capture and understand the large-scale cellular context of many biological systems in humans and several model organisms (3,4). Fortunately, due to community-wide norms and funding requirements, nearly all of the resulting transcriptomes have been deposited in publicly-available repositories (5–8). For example, as of 29 January 2020, there are >2 million human microarray samples from >24k datasets along with about half as much human RNA-seq data (>583k samples from ∼12k datasets) contained in the NCBI Gene Expression Omnibus (GEO) database (7,8).

The purpose of these publicly-available data is to enable other researchers to use published datasets to reproduce original findings, reuse datasets in new ways to answer new questions (9), or combine thousands of datasets to build integrative models (10) towards precision medicine (11). However, a major hurdle in realizing these goals is the fact that microarray profiles have been measured using a number of different platforms that each measure a different number of pre-defined genes (ranging from a few hundred genes to ∼20k genes). For instance, the most popular genome-scale platform *Affymetrix Human Genome U133 Plus 2.0 Array* (GEO ID: *GPL570*) accounts for only 22% of the >2 million samples. The next most popular *Affymetrix Human Genome U133A Array* (GEO ID: *GPL96*) accounts for another 11% of the samples, but only covers <12k genes. Therefore, it is a significant challenge to gain insights about the full complement of genes in the human genome across the diversity of biological samples and unique experimental conditions in existing microarray data.

In addition to these researcher-submitted microarray datasets, concerted effort has also been put into defining a reduced set of genes that can be measured and then be used to accurately recover the expression of all the other genes (12,13). The most prominent example of this effort is the Library of Integrated Network-Based Cellular Signatures (LINCS) microarray program (14), which has shown that measuring 978 ‘landmark’ genes, costing only $5 per sample (15), is sufficient to then use to impute the expression of all other (tens of thousands of) genes. There are currently 1.3 million microarray samples in the LINCS data repository capturing the effect of numerous chemical and genetic perturbations on gene expression (14).

**Figure 1.**
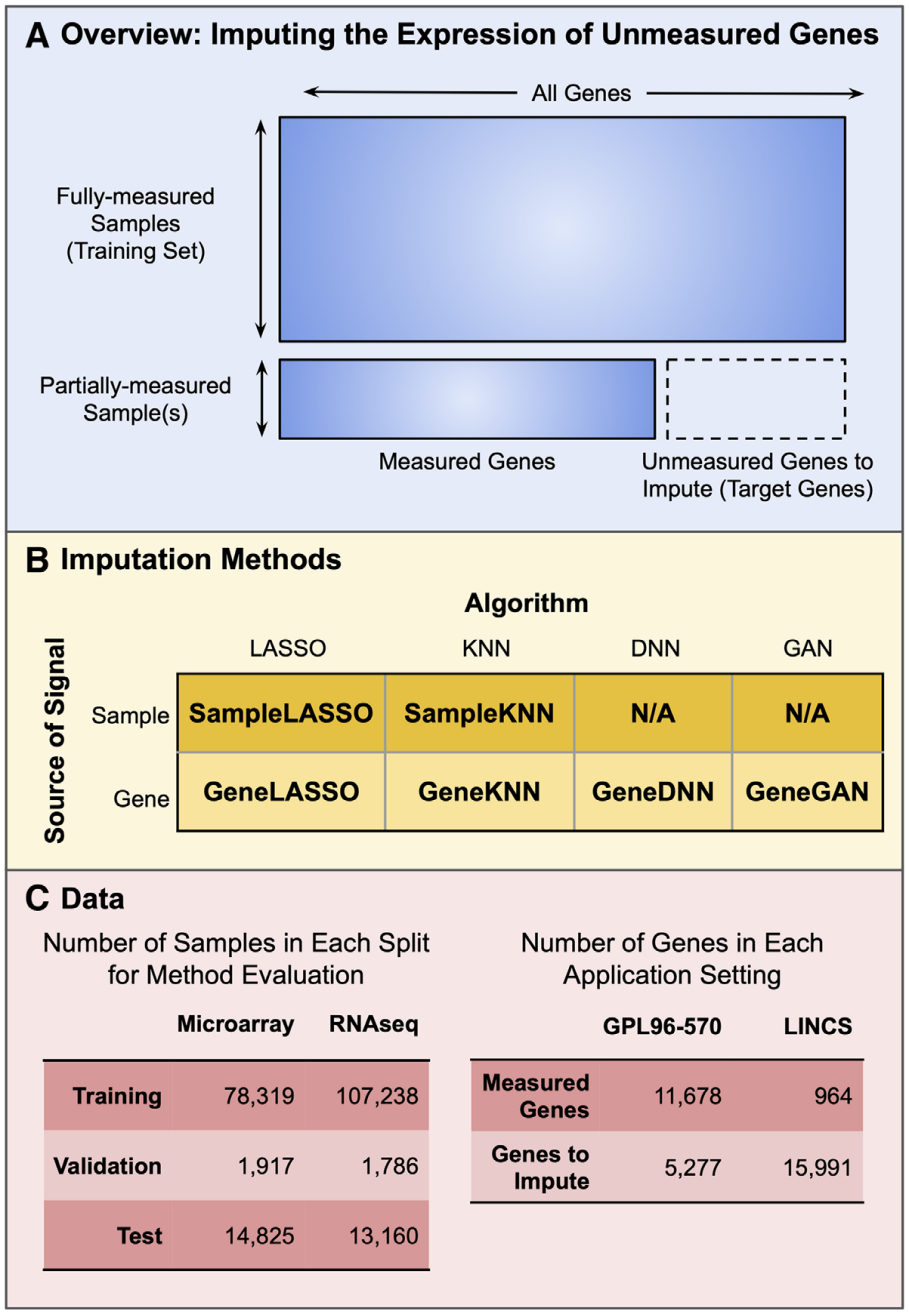
**Overview of gene expression imputation**. (**A**) Schematic of the problem of ‘imputing the expression of unmeasured genes’. A training dataset is used to fill in the expression values of genes from partially-measured samples. The six methods (**B**) and summary of the data (**C**) used in this study. The methods are named using a combination of the machine learning algorithm and biological signal that the method uses.

With either of these massive data collections—the >1 million public transcriptomes from various microarray platforms or the 1.3 million LINCS profiles—restricting analysis and integration to the measured genes common to all platforms/samples will result in a tremendous loss of valuable data. Therefore, effectively leveraging the full data compendia on a genome-scale necessitates computational methods that can use the expression levels of the measured genes in a *partially-measured microarray sample* to impute the expression of all *unmeasured genes* in that sample to reconstitute a complete transcriptome (Figure 1A). A few previous studies have indeed proposed methods to solve this problem in various settings. Sparse regression models that use *gene-gene correlation signals* have been shown to be effective in imputing gene expression in samples from the GPL96 (<12k genes) microarray platform based on samples from the GPL570 (whole-genome) platform (16). Others have developed methods that use gene correlations based on low-rank regression (17) and deep neural networks (18,19), specifically for the LINCS dataset. These methods rely on training machine learning models that map the relationship between fixed sets of measured and unmeasured genes in a specific setting, be it sparse gene-based regression for the GPL570-96 setting (16) or deep learning for the LINCS setting (18,19). Methods have also been proposed to address the problem of identifying the best reduced set of genes to measure to enable subsequent imputation of all other genes, again within the scope of specific large datasets (12,13,20). However, all these methods lack the flexibility for broad adoption since public datasets come from many different expression-profiling technologies, with each measuring the expression of a different subset of genes in the genome. All current methods are hard to adapt for imputing unmeasured gene-expression in an arbitrary experiment since they require training completely new models for every microarray platform (or every new reduced gene set design), which, in turn, requires very large datasets for model-training.

Leveraging gene–gene correlations in data was an important component of gene expression imputation that focussed on the related-yet-distinct ‘missing value’ problem (Supplementary Figure S1), concerned with recovering the expression values of individual genes that were lost *within a single dataset* due to arbitrary technical error in samples, i.e. filling arbitrary empty cells within a larger data matrix (21–23). Many methods have been proposed to tackle this problem (24–30), and, in general, imputing missing values has been shown to improve downstream tasks such as clustering, classification, co-expression network building, and differential expression (31–34). Although these seminal works on the missing-value problem guide imputation methods today, the fact that we can now leverage information from >100k samples at a time to improve the imputation of unmeasured genes requires a rethinking of imputation strategies. Thus, it is critical that new imputation methods select only the most relevant samples to the target sample, as gene–gene correlations change across different biological contexts (35).

In this study, we demonstrate that using a sparse-regression method that leverages information from the most similar samples provides more accurate predictions than other methods while also providing a highly interpretable underlying model. Current state-of-the-art imputation methods train machine learning models that capture the relationship of the predetermined set of measured genes to each predefined unmeasured gene (or set of unmeasured genes). Then, during imputation in an expression sample with the same measured/unmeasured genes, these methods use the pretrained models to impute the expression of the unmeasured genes. We propose a variant of this approach that we call *SampleLASSO* in which, for every new expression sample to be imputed, a new sparse regression model is trained on-the-fly that captures the relationship of this expression sample to all others in the training set based on the genes measured in that given sample. We compare our method to four other imputation methods based on three different algorithms—*k*-nearest neighbors, regularized linear regression, and deep neural networks—that leverage gene-gene or sample-sample relationships (Figure 1B). All these methods are evaluated on imputation within the same-technology (microarray and RNA-seq), as well as using RNA-seq data to impute microarray data. The evaluation is carried out for two different, practical unmeasured gene settings: (i) a high number of measured genes and low number of unmeasured genes (GPL96–570 gene subset) and (ii) a small number of measured genes and large number of unmeasured genes (LINCS gene subset) (Figure 1C). Extensive evaluations using multiple accuracy metrics within a rigorous temporally-split and dataset-preserving scheme showed that the flexible sample-based imputation method (*SampleLASSO)* always shows competitive performance with the best performing methods, and in particular is always the best performing method when using data from one expression platform to impute data in another expression platform. We also demonstrate the biological interpretability of this method by showing that, for imputing a given sample from a certain tissue, the *SampleLASSO* model automatically up-weights training samples from the same tissue type.

## MATERIALS AND METHODS

### Data

We used gene expression data from both microarray and RNA-seq technologies. For the microarray data, we downloaded all human samples from the *Affymetrix Human Genome U133 Plus 2.0 Array* from NCBI GEO (8) as raw CEL files and performed background subtraction, quantile transformation, and summarization using fRMA (36) based on a custom CDF (37) mapping probes to Entrez gene IDs. This yielded 108 205 samples with 19 702 genes. For the RNA-seq data, we downloaded all 133 776 TPM-normalized human samples from *ARCHS4* (5), and further processed the data by converting ENST IDs to Entrez gene IDs for only the genes found in the microarray data. Genes that could not be mapped this way were discarded from both microarray and RNA-seq data. This yielded a total of 16 955 genes. Finally, the RNA-seq data was then transformed using the inverse hyperbolic sine (archsinh) function.

We additionally downloaded preprocessed data for 10 gene expression platforms contained in the SEEK database (38). For each gene expression platform in SEEK, we constrained the data to only include genes common to all experiments associated with the platform, and if an experiment contained any samples with missing values, that entire experiment was removed. More information on data processing is provided in Section 1.2 of the Supplemental Material.

### Validation scheme

#### Subsetting genes

To evaluate the imputation methods, we chose to split genes into measured and unmeasured sets to represent two very different practical scenarios (Figure 1C). First, we considered the situation in which we have a large number of measured genes that we could use to impute a smaller number of unmeasured genes. This scenario presents itself in the problem of using the 11 678 genes measured in the older human microarray platform *Affymetrix Human Genome U133A Array* (i.e. GPL96) to then impute the expression of an additional 5 277 genes that are only present in the newer genome-scale platform *Affymetrix Human Genome U133 Plus 2.0 Array* (i.e. GPL570) (16). This gene-split is referred to as the *GPL96–570 gene subset* in this work. Second, we considered the situation in which we have a small number of measured genes that we could use to impute a large number of unmeasured genes. For this scenario, we used 964 ‘landmark’ genes from LINCS as the measured genes to impute the expression of all the other genes in the genome-scale *Affymetrix Human Genome U133 Plus 2.0 Array* (15 991 unmeasured genes) (14,18). This gene-split is referred to as the *LINCS gene subset* in this work.

To determine the set of unmeasured genes to impute in the data from multiple platforms downloaded from the SEEK database, we first found genes that were both common among all ten platforms in SEEK and also contained in the 16 955 genes from the *Affymetrix Human Genome U133 Plus 2.0 Array* and *ARCHS4* data we processed. We used a file that contains the number of PubMed articles a gene was mentioned in (39) to break this set of genes up into three categories; highly-, moderately-, or scarcely-studied genes. We then chose 30 genes from each of these three sets, resulting in the same set of 90 unmeasured genes to be imputed for all the platforms contained in the SEEK database. Each platform in SEEK had a different set of measured genes which was determined by using genes common between that platform and the 16 955 genes from the *Affymetrix Human Genome U133 Plus 2.0 Array* and *ARCHS4* data we processed, but excluded any gene in the set of the 90 genes used in the unmeasured set (Supplementary Table S1).

#### Splitting samples

We divided the expression samples into training, validation, and testing sets. The training data was used to fit the models, the validation data was used for hyperparameter tuning, and the testing data was used in the final evaluations of the models (shown in all the figures in the main text). To mitigate data leakage, we ensured that entire datasets were assigned to splits, thus keeping all expression samples from the same experiment (dataset) together in the same split. The data was also temporally split, with the oldest expression samples being placed in the training and validation sets, and the newest samples going into the test set. To speed up hyperparameter tuning, which consisted of training >500 000 individual models, we further subsetted the validation set by taking 10% of the expression samples from each experiment in the full validation set (or at least two expression samples, if the number of samples in an experiment was <20) (see Section 1.2 in Supplemental Material and Supplementary Figure S2).

For the analysis of imputing data from the SEEK database, we used the same training data for every platform: the training data set from the *Affymetrix Human Genome U133 Plus 2.0 Array* described above. For each platform in SEEK, we generated a test set from 90 randomly selected samples contained in that platform to ensure that both sample- and gene-based methods had the same number of examples to predict during testing.

For all imputation methods, we standardized each feature in the training set by subtracting the mean and dividing by the standard deviation of the given feature. Correspondingly, each feature in the validation and test sets was standardized using the mean and standard deviation obtained from the training set.

### Imputation methods

In this study, we evaluated imputation methods using four distinct machine-learning algorithms: *k*-nearest neighbors (*KNN*), least absolute shrinkage and selection operator (*LASSO)*, a fully-connected feedforward deep neural network (*DNN*), and a conditional generative adversarial network (*GAN*).

*KNN* is a machine learning algorithm that predicts the target variable for every new example based on the target variables of the *k* most similar examples in the training data. To impute a given target variable, we used a weighted average of the measured target variable from the *k* most similar examples based on Euclidean distance, with the weight equal to the inverse of the distance. KNN imputation is a widely-used imputation method for gene expression and provides a strong baseline (12,18,22,24).

*LASSO* is a linear regression method in the family of least-squares optimizers (40). LASSO builds a sparse model for a given target variable using the following cost function:

(1) $$\begin{equation*}mi{n_\beta }\frac{1}{{2N}}\left| {\left| {X\beta - y} \right|} \right|_2^2 + \alpha {\left| {\left| \beta \right|} \right|_1}\end{equation*}$$

where *N* is the number of training examples, $\beta$ is the vector of learned parameters, $X$ is the training data, $y$ is the target variable, $\alpha$ is the hyperparameter that determines the extent of L1-regularization (${| {| \beta |} |_1}$). L1-regularization prevents overfitting by setting many of the elements of $\beta$ to 0. While a variety of least-squares optimizers have been applied to the gene expression imputation problem (22,25,41,42), LASSO is the method most suited when the number of features is large (12,18,43). In this study, we used the KNN and LASSO implementations contained in the Python package *scikit-learn* (44).

A *DNN* is a multi-layer feedforward neural network with bespoke architectures designed for each machine learning task. A *GAN* is a deep neural network consisting of two main parts: a ‘generator’ that generates imputed values and a ‘discriminator’ that attempts to discriminate between imputed values and the ground truth expression values. For both the *DNN* and *GAN* models, we used architectures from recently published works that evaluated the utility of these models for imputing gene expressions using the LINCS landmark genes; namely the *D-GEX* model for *DNN* (18) and the *GGAN* model for *GAN* (19). The *DNN* and *GAN* models were trained using Nvidia Tesla k80 GPUs and implemented using the Python package *Keras* (45) with a *Tensorflow* backend (46). For more information on the deep learning methods, see Section 1.3 of the Supplemental Material.

We used these four algorithms—KNN, LASSO, DNN, GAN—to leverage two distinct types of signals—gene–gene similarities (across samples) and sample–sample similarities (across genes)—for imputing the expression of unmeasured genes in a new partially-measured sample, resulting in six methods referred to in this study as *SampleKNN*, *GeneKNN*, *SampleLASSO* (proposed here), *GeneLASSO*, *GeneDNN* and *GeneGAN*. For intuitive, pictorial schematics of the methods, see Supplementary Figures S3-S7 in Section 1.3 of the Supplemental Material.

*SampleKNN* and *GeneKNN* are the most straightforward and popular implementation of KNN for gene expression imputation. For a new partially-measured expression sample to be imputed, *SampleKNN* works by first finding the *k* most similar samples in the training set based on the expression of all measured genes, and then imputing the expression of each unmeasured gene with the weighted average of that gene's expression in the most similar training samples. Thus, the major biological signal used is the similarity between samples (across genes). Conversely, *GeneKNN* works on a gene-by-gene basis. For each gene that is missing (unmeasured) in a new sample, the method first finds the *k* measured genes most similar in their expression pattern across all the samples in the training set, and then imputes the expression of the unmeasured gene with the weighted average of the expression of those *k* genes in that new sample. Thus, the major biological signal used is the similarity between genes (across samples).

*GeneLASSO* is the traditional, widely adopted means of implementing LASSO for gene expression imputation. Here, using the fully-measured training set, a separate sparse regression model is trained for each unmeasured gene, to predict its expression based on a linear combination of all the measured genes. Then, given a new partially-measured sample, the expression of every unmeasured gene is imputed using that gene's pre-trained model, with the predicted expression being equal to the sum of the expression of the measured genes in the new sample weighted by the model coefficients. Akin to *GeneKNN*, the main source of biological signal for *GeneLASSO* is gene–gene expression similarities. As an alternative to *GeneLASSO*, which requires information about which genes are unmeasured in a new sample and a pre-trained model for each of those genes, in this study, we propose a simple alternative called *SampleLASSO*. Given a new partially-measured sample, *SampleLASSO* builds a single model on-the-fly that predicts that sample's expression profile based on a sparse linear combination of all the samples in the training set only using the subset of genes measured in the new sample. Here, for every sample to be imputed, the coefficients of the trained model in essence finds the relationship of that sample to all samples in the training set. Then, all the unmeasured genes are imputed using this trained sample-specific model. The main source of biological signal in SampleLASSO, thus, comes from sample similarities. We note a method similar to *SampleLASSO* has been reported before (called *LS_array*) (25). However, the implementation of that method was focused on the missing value problem and has never been applied to the unmeasured gene problem.

*GeneDNN* and *GeneGAN* use deep learning to predict the expression of (a fixed set of) unmeasured genes using a single model that captures complex, nonlinear relationships between the unmeasured genes and the measured genes using the training set. Thus, the main biological signal in *GeneDNN* and *GeneGAN* is also from gene-gene relationships.

### Hyperparameter tuning

For both KNN and LASSO, there is only one hyperparameter that needs to be tuned; *k:* the number of most similar training examples to consider in KNN and $\alpha$: the parameter that sets the strength of the L1-regularization term in LASSO. Although there are many hyperparameters to tune in a DNN or GAN, we fixed most parameters based on optimal values found in the previously published works (18,19), and just tuned the optimizer and learning rate. Hyperparameter tuning was done using the validation set data (Section 1.4 in Supplemental Material, Supplementary Figures S9–S15, and Supplementary Table S2), and the optimal hyperparameters were then used for final evaluation using the test set. We note that, for *GeneDNN and GeneGAN*, we used all the ∼13k samples in the validation set for hyperparameter tuning as: (i) this most closely mimics the setup in the original papers and (ii) deep learning models additionally use the validation set to determine which epoch (i.e. how many passes through the data the model goes through) yields the best model. For the analysis using the SEEK data, the best hyperparameter across all the tasks and gene subsets using the *Affymetrix Human Genome U133 Plus 2.0 Array* and *ARCHS4* data was used for all ten platforms. For more information on hyperparameter tuning, see Section 1.4 of the Supplemental Material.

### Evaluation metrics

A commonly used metric for evaluating gene expression imputation methods is Normalized Root Mean Square Error (NRMSE). The NRMSE for a gene (${g_i})$ is given by:

(2) $$\begin{eqnarray*}NRMSE\left( {{g_i}} \right)\ = \ \ \frac{{RMSE\left( {{g_i}} \right)}}{{Mean\left( {{g_i}} \right)}} = \frac{{\sqrt {\mathop \sum \nolimits_{j = 1}^S {{\left( {{{\hat{g}}_{i,j}} - {g_{i,j}}} \right)}^2}/S} }}{{\mathop \sum \nolimits_{j = 1}^S {g_{i,j}}/S}}\nonumber\\ \end{eqnarray*}$$

where *RMSE* is the root mean square error, $S$ is the number of samples, and ${\hat{g}_{i,j}}$, ${g_{i,j}}$ are the imputed and real expression values, respectively, for the *i*th gene in the *j*th sample.

In addition to NRMSE, we also report evaluation results using the Spearman correlation coefficient and the Mean Absolute Error (MAE) in Section 2.2 of the Supplemental Material.

### Interpreting *SampleLASSO* models

We evaluated the interpretability of *SampleLASSO* models by examining if the $\beta$-coefficients of a model trained for a particular sample recapitulated that sample's tissue-of-origin by assigning high positive $\beta$ values to samples in the training set from the same tissue relative to samples from all other tissues.

Specifically, for a given target sample *s* that we built a *SampleLASSO* model for, we calculated a *z*-score, ${z_{s,T}}$, for each tissue $T$ in this annotated set based on the $\beta$ values of training samples from that tissue:

(3) $$\begin{equation*}{z_{s,T}}\ = \frac{{\left( {\mathop \sum \nolimits_{j:tissue\left( j \right) = T}^{} {\beta _j}} \right)/\left| T \right|\ - \ {\mu _s}}}{{{\sigma _s}/\sqrt {\left| T \right|} }}\end{equation*}$$

where $| T |$ is the number of labeled samples for tissue $T$, ${\beta _j}$ is the value of the $\beta$-coefficient from the *SampleLASSO* model for the *j*th sample, and ${\mu _s}$and ${\sigma _s}$ are the mean and standard deviation of the $\beta$-coefficients of all samples in the training set that have any tissue label.

To perform this analysis we used a large set of expression samples that were manually-curated to their tissue-of-origin (>15k samples) (47). However, due to the initial temporal split of the data into training, validation, and test sets, all the labeled expression samples were in the original training set. Hence, just for this interpretability analysis, we separated out a subset of the tissue-labeled samples in the original training set into a new manually-curated test set. We created this subset so that it spanned six tissues that were sufficiently diverse and were labeled to at least 10 samples from at least three different datasets in both the training and the test sets. This resulted in the new test set having 222 expression samples from 29 different datasets. The full training set consisted of all samples from the original training set, expect we removed any sample that was part of the same experiment as any test set sample. Of these 77 893 training samples, 11 618 samples had a manually curated tissue label, with 4397 expression samples from 120 different datasets having a label pertaining specifically to the six tissues the analysis was carried out for (Table S4). To calculate ${\mu _s}$ and ${\sigma _s},$ we used any sample that had a tissue label, regardless of tissue type, allowing us to use 11 618 samples for these calculations. A *SampleLASSO* model was trained for each manually labeled test sample and used in the *z*-score analysis above (Equation 3).

## RESULTS

In this study, we compare imputation methods that use four distinct machine learning algorithms: least absolute shrinkage and selection operator (LASSO), k-Nearest Neighbors (KNN), a fully-connected feedforward neural network (DNN), and a conditional generative adversarial network (GAN) (Figure 1B). Combining these algorithms with the source of the data signal—gene–gene or sample–sample relationships—resulted in six imputation methods: *SampleLASSO*, *GeneLASSO*, *SampleKNN, GeneKNN, GeneDNN* and *GeneGAN*. These methods are evaluated in two settings with different sets of unmeasured genes (Figure 1C): (i) the GPL96–570 gene subset, which uses a relatively large number of genes (∼11 000) to impute the expression of a smaller number of genes (∼5000) and (ii) the LINCS gene subset, which uses a relatively small number of genes (∼1000) to impute the expression of a large number of genes (∼16 000). We consider imputation using data from the same technology (using microarray to impute microarray, and RNA-seq to impute RNA-seq) as well as across technologies (using RNA-seq to impute microarray data). We evaluate methods using both the scale-free regression error metric (normalized root mean squared error; NRMSE), as well as Spearman correlation and mean absolute error. We also evaluate the performance of the imputation methods on data from ten additional gene expression platforms downloaded from the SEEK database (38). Lastly, we examine the model coefficients learned by *SampleLASSO* for biological interpretability.

**Figure 2.**
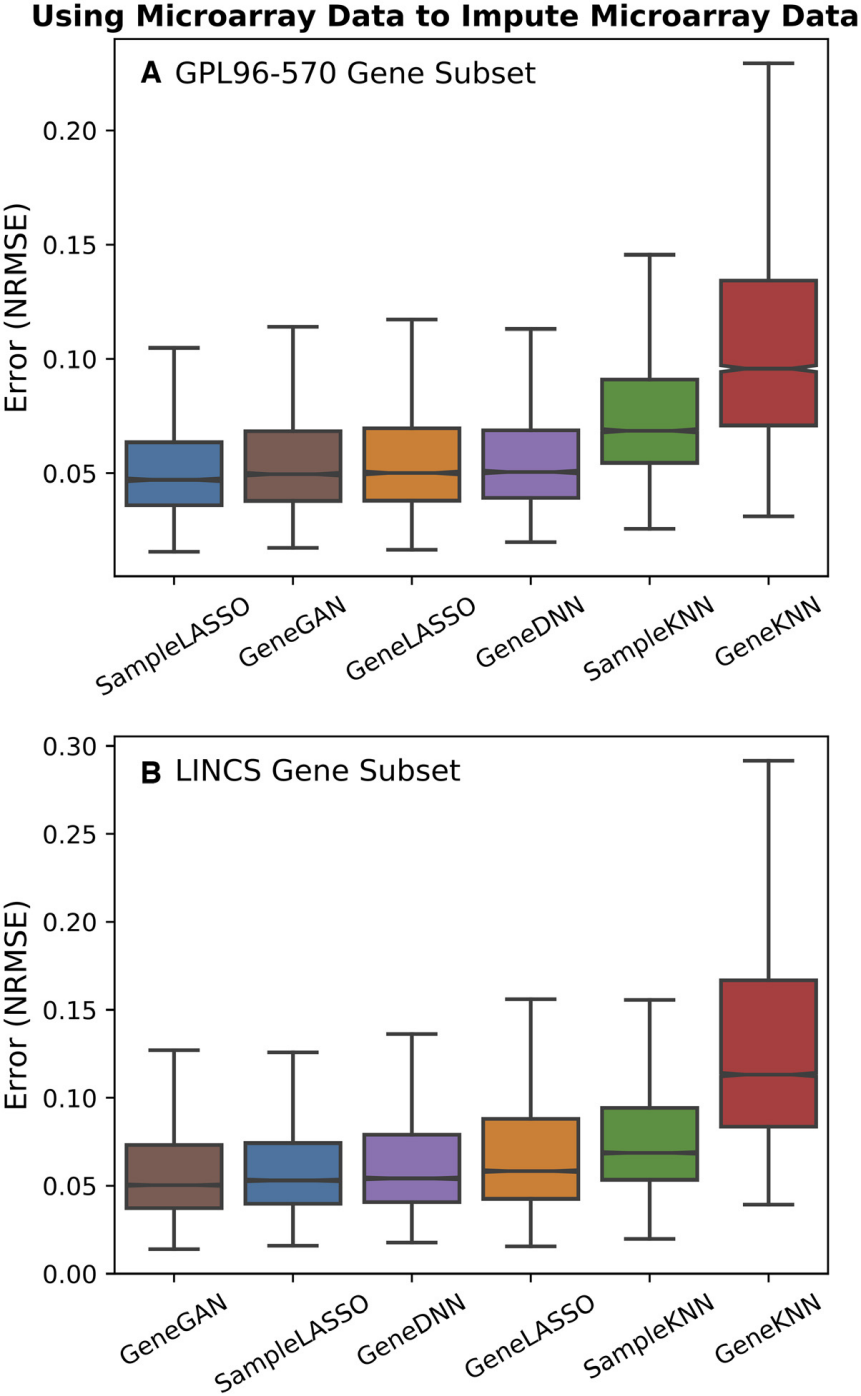
**Performance of imputation methods for microarray data**. Boxplots showing the performance of the six imputation methods (*SampleLASSO*, *GeneGAN*, *GeneDNN*, *GeneLASSO*, *SampleKNN*, *GeneKNN*) across two gene subsets (**A**: GPL96-570 and **B**: LINCS), trained and imputed on microarray data. The evaluation metric is NRMSE, with lower values indicating better performance, and the methods are ordered by the median value.

We first evaluated the performance of the six imputation techniques using microarray data to impute microarray data (the microarray data here all comes from the *Affymetrix Human Genome U133 Plus 2.0 Array*) (Figure 2). For the GPL96–570 gene subset task, *SampleLASSO* is the best performing model, whereas for the LINCS gene subset, *GeneGAN* is the best performing method. For both gene subsets, both KNN methods perform relatively poorly. For the GPL96–570 gene subset, *SampleLASSO* outperforms *GeneGAN* 92% of the time, *GeneLASSO* 91% of the time, *GeneDNN* 95% of the time, *SampleKNN* 100% of the time, and *GeneKNN* 100% of the time. For the LINCS gene subset, these percentages are 25%, 91%, 77%, 98% and 100%, respectively. Statistical tests and effect sizes between *SampleLASSO* and the other methods can be found in Supplementary Table S3.

**Figure 3.**
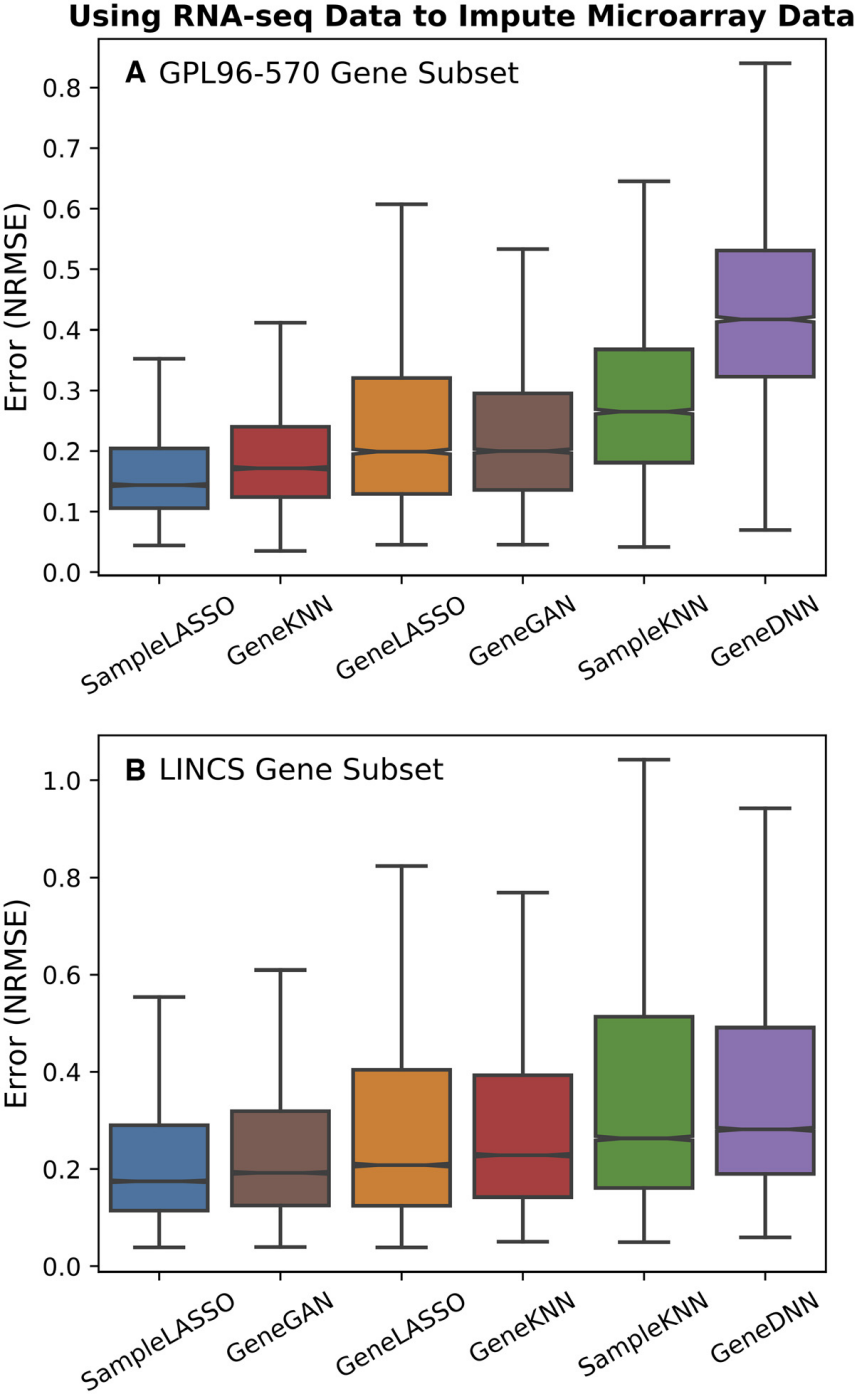
**Performance of imputation methods for cross-technology imputation**. Boxplots showing the performance of the six imputation methods (*SampleLASSO*, *GeneGAN*, *GeneDNN*, *GeneLASSO*, *SampleKNN*, *GeneKNN*) across two gene subsets (**A**: GPL96-570 and **B**: LINCS) using RNA-seq data to impute microarray data. The evaluation metric is NRMSE, with lower values indicating better performance, and the methods are ordered by the median value.

Although microarray platforms like the *Affymetrix Human Genome U133 Plus 2.0 Array* are able to quantify the expression of nearly all protein-coding genes, RNA-seq technology enables the quantification of nearly all cellular transcripts from both annotated and unannotated genes. Hence, it would be valuable to use RNA-seq data to predict the expression of genes missing in microarrays, enabling (i) re-analysis of novel genes in experimental settings captured in the vast number of microarray datasets and (ii) joint analysis and integration of RNA-seq and microarray data based on a common set of genes. We evaluated the performance of using *ARCHS4* RNA-seq data to impute *Affymetrix Human Genome U133 Plus 2.0 Array* microarray data using the GPL96–570 and LINCS gene subsets (Figure 3). *SampleLASSO* is the best performing method for both gene subsets. For the GPL96–570 gene split, *SampleLASSO* outperforms *GeneGAN* 71% of the time, *GeneLASSO* 71% of the time, *GeneDNN* 91% of the time, *SampleKNN* 80% of the time, and *GeneKNN* 70% of the time. For the LINCS gene split, these percentages change to 56%, 62%, 76%, 76% and 84%, respectively. The Wilcoxon ranked-sum test between *SampleLASSO* and the other methods showed that the performance increase of *SampleLASSO* was always statistically significant (*P*-value << 0.001).

**Figure 4.**
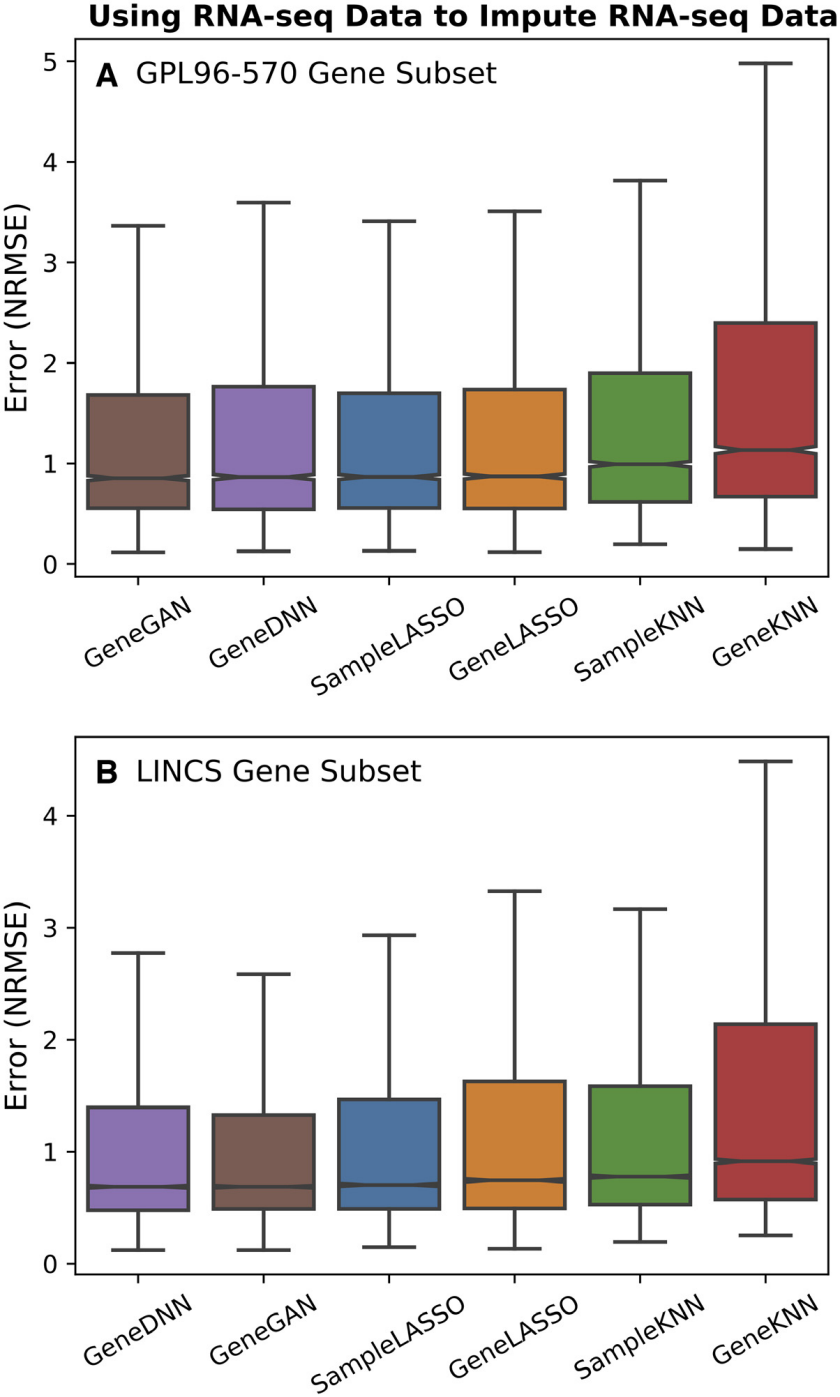
**Performance of imputation methods for RNA-seq data**. Boxplots showing the performance of the six imputation methods (*SampleLASSO*, *GeneGAN*, *GeneDNN*, *GeneLASSO*, *SampleKNN*, *GeneKNN*) across two gene subsets (**A**: GPL96-570 and **B**: LINCS) using RNA-seq data to impute RNA-seq data. The evaluation metric is NRMSE, with lower values indicating better performance, and the methods are ordered by the median value.

We also evaluated the methods using RNA-seq data to impute RNA-seq data (the RNA-seq data here all comes from the *ARCHS4* database) (Figure 4). We note that using RNA-seq data to impute RNA-seq data does not have as many obvious applications as the microarray setting since RNA-seq technologies do not require pre-determining a set of genes to measure, and thus have high gene coverage. For this task, one of the deep learning methods is the best performing method for both gene subsets. For the GPL96–570 gene split, *SampleLASSO* outperforms *GeneGAN* 40% of the time, *GeneLASSO* 41% of the time, *GeneDNN* 39% of the time, *SampleKNN* 93% of the time, and *GeneKNN* 98% of the time. For the LINCS gene split, these percentages change to 31%, 62%, 13%, 84% and 100%, respectively. Statistical tests and effect sizes between *SampleLASSO* and the other methods can be found in Supplementary Table S3. For all three imputation tasks and both gene subsets, we find similar results when measuring imputation accuracy using Spearman correlation and mean absolute error (Supplementary Figure S16–S18), except for the RNA-seq to microarray imputation task on the LINCS gene subset, where for Spearman correlation *GeneGAN* and *GeneLASSO* both outperform *SampleLASSO*.

When imputing gene expression values from one platform or technology to another, the imputation error stands to be lowered by jointly normalizing the data in an appropriate way before imputation. This is not an easy task as the distribution of the unmeasured genes for the samples to be imputed is inherently not known. However, to get a sense of how much even applying basic normalization helps the RNA-seq to microarray imputation task, we used the known ground truth values for the unmeasured geneset to jointly quantile normalize all of the data together; a similar normalization strategy was applied in recent imputation studies (18,19). Performing this joint quantile normalization of the two datasets results in the expected decrease in imputation error over the no-normalization case (Supplementary Figure S19).

We additionally looked at the performance of the imputation methods as a function of the mean expression and variance of the unmeasured genes. For each unmeasured gene, we placed it into a low, medium or high bin based on its mean expression and variance in the context of the known expression values across all the samples (Supplementary Figures S20–S25). Although the relative performance of the imputation methods does not change too much when looking at the different categories of mean expression and variance, it can be seen in some cases that *SampleLASSO* performs particularly well for genes with low mean expression and high variance, which is the hardest category of genes to impute.

**Figure 5.**
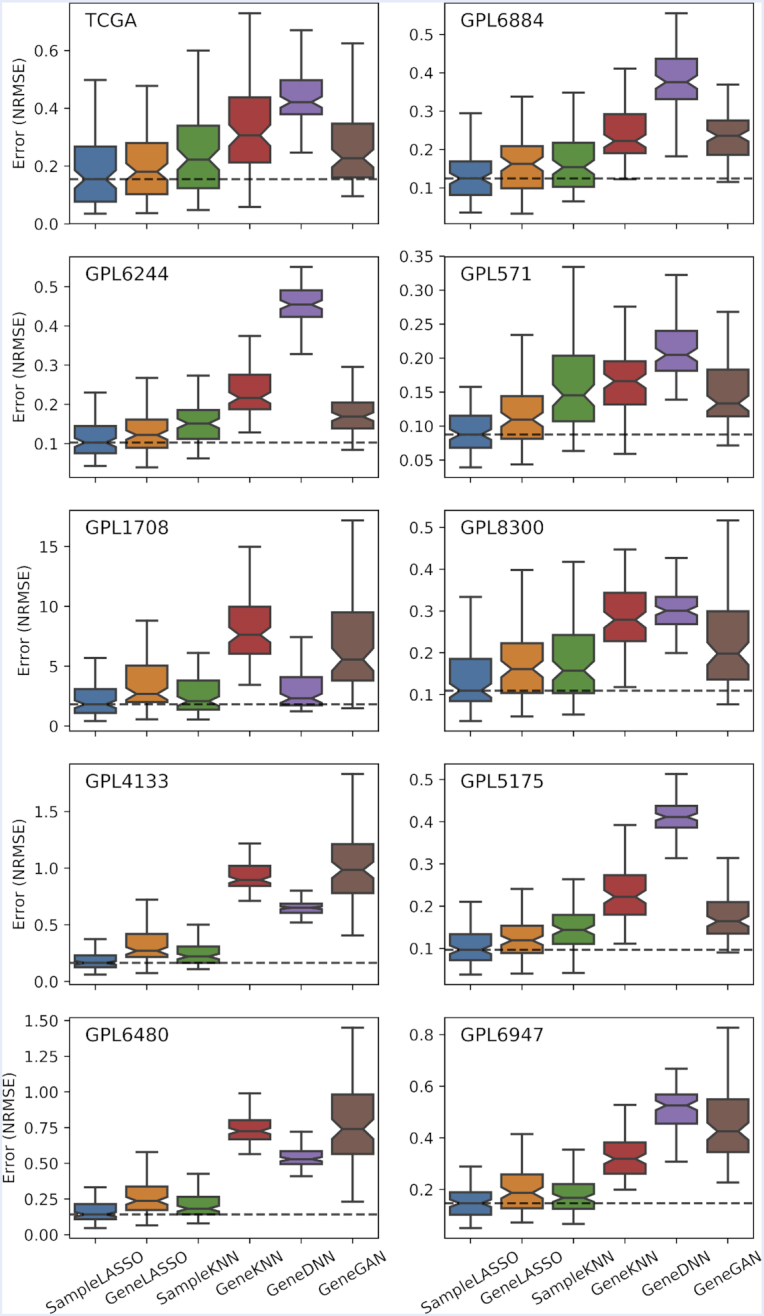
**Performance of imputation methods on ten expression platforms**. Boxplots showing the performance of the six imputation methods (*SampleLASSO*, *GeneGAN*, *GeneDNN*, *GeneLASSO*, *SampleKNN*, *GeneKNN*) across ten gene expression platforms. The evaluation metric is NRMSE, with lower values indicating better performance. The horizontal dashed line indicates the median value of *SampleLASSO*.

As stated before, since there are millions of microarray samples from dozens of gene expression platforms, each with a different subset of measured genes, it is critical that a good imputation method can accurately impute gene expression values in all these different settings. To evaluate this practical application, we tested the six imputation methods on their ability to impute gene expression values in data from ten platforms (obtained from the SEEK database) (38) (Figure 5, Supplementary Figure S26). We note that this analysis of imputing expression values across many different platforms has not been conducted in any of the recent studies on gene expression imputation.

For this analysis, we choose a set of 90 genes common to all platforms, and containing highly-, moderately-, and scarcely-studied genes, as the set of unmeasured genes. For all ten platforms, we used the same 78 319 training samples from the *Affymetrix Human Genome U133 Plus 2.0 Array* data. For each of the 10 platforms, the set of measured genes was determined by taking the genes common to a given platform and the *Affymetrix Human Genome U133 Plus 2.0 Array* data, excluding any gene that was in the set of 90 unmeasured genes. It can be seen that *SampleLASSO* is the best performing method across all ten platforms, whereas both deep learning models perform quite poorly. For more information on the analysis using the SEEK data, see Section 2.5 of the Supplemental Material.

Although reducing the error of imputation methods is of the utmost importance, the applicability of an imputation method is also greatly increased if the model offers biological insight into how the predictions were generated. For instance, *SampleKNN* offers immediate interpretability via the biological/experimental contexts of the *k*-nearest training samples picked by the method, however it is not very accurate in imputing unmeasured genes. Since we devised *SampleLASSO* with this desirable property in mind, we tested if this new method also offers biological interpretability in addition to providing very accurate imputation. Since gene expression samples have clear signals pertaining to their tissue-of-origin (35), we focused on testing if the *SampleLASSO* model trained for a new sample from a particular tissue up-weighted training samples from that same tissue relative to training samples from other tissues.

**Figure 6.**
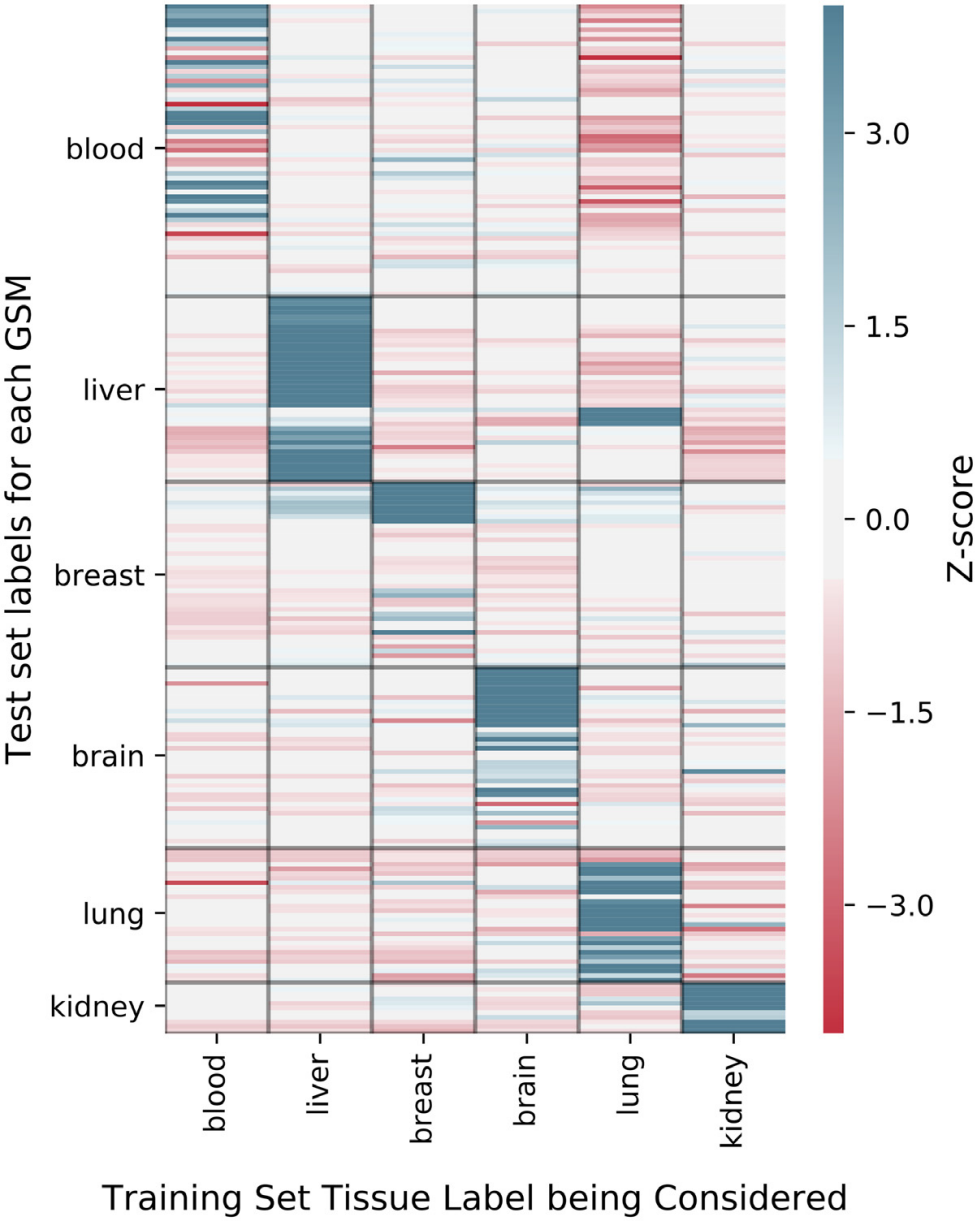
***SampleLASSO*** **captures biologically relevant information**. Biological interpretability was evaluated using expression samples labeled with tissue-of-origin to determine if *SampleLASSO* up-weights training samples of the same tissue as the test sample in the sparse model. The rows represent the 222 tissue-labeled samples (GSMs) in the test set. The black horizontal lines separate test samples from different tissues. The columns correspond to the tissue type of the training samples. The colors of the heatmap represent the *z*-scores. Calculated per test sample (row), the *z*-score per tissue (column) corresponds to the normalized aggregate of the model coefficients of all the training samples from that tissue. See Materials and Methods for more details. The diagonal blocks correspond to the case where the *z*-scores were calculated for the same tissue type as the tissue-of-origin of the test sample.

To perform this analysis we used a large set of expression samples that were manually labeled to their tissue-of-origin (47). Due to limited labeled tissues and their representation in our data, the analysis was restricted to six sufficiently-diverse tissues that had at least 10 samples from at least three different datasets in both the training set and the test set (Supplementary Table S4). Then, for each test sample, we trained a SampleLASSO model and used the model coefficients ($\beta$-coefficients) to calculate a *z*-score for each of the six tissues that represents the aggregate $\beta$-coefficients corresponding to training samples just from that tissue relative to the background distribution of $\beta$-coefficients of all labeled training samples (not just samples labeled from the six tissues). Thus, a large positive *z*-score for a particular tissue means that samples from that tissue were more informative than others. See *Materials and Methods* for more details. Implementing this analysis on the entire labeled test set, we observe that for most test samples, the strongest signal captured by *SampleLASSO* comes from training samples from the same tissue as the sample being imputed (Figure 6, Supplementary Figure S27).

To aid in reproducibility, we have publicly released all the processed data that we used on Zenodo (https://doi.org/10.5281/zenodo.3971092) as well as all the code to re-generate the results and figures on GitHub (https://github.com/krishnanlab/Expresto). In addition, we also provide a user-friendly function that performs imputation using *SampleLASSO* given a file of expression data. The function will return the imputed expression values as well as a list of the most utilized samples from the training set (see Supplementary Table S5 for an example).

## DISCUSSION

In this study, we propose a simple, new method termed *SampleLASSO* for imputing the expression of unmeasured genes in partially-measured gene-expression profiles. The fundamental benefit of imputing unmeasured genes in samples from a partially-measured platform is the ability for researchers to reanalyze the data from all these samples on a genome-scale. Thus, important common tasks such as building co-expression networks, differential expression analysis and gene set enrichment will now include the full complement of genes in the genome. This benefit is particularly evident for data in the LINCS database, where samples only measure expression from ∼1000 genes. An additional major benefit comes from the ability to perform integrative analysis of data from multiple platforms with maximum gene coverage. This increase in coverage is critical for data-integration efforts or big data-driven machine learning models that pull together data across many platforms. These efforts will be severely stifled when they are forced to only include genes common to all platforms, resulting in a huge loss of information. For example, for combining all the data in the SEEK database, with imputation, we can build a gene-expression set that contains the expression of 19 124 genes (the number of genes in the platform with the highest gene coverage), whereas if we only included genes common to all the platforms, that expression set would only contain the expression of 6017 genes.

*SampleLASSO* is a sparse regression model that trains a machine learning model on-the-fly for every expression profile that needs to have expression values imputed. We demonstrate that *SampleLASSO* is a highly accurate method for imputing unmeasured genes based on an extensive evaluation on three different imputation tasks (within and across technologies), two imputation settings that differ in the number of measured genes by an order of magnitude, and across multiple gene expression platforms. *SampleLASSO*’s strength comes from its ability to effectively leverage information from samples from the same biological context.

In addition to helping estimate the performance of imputation methods, our analyses in different imputation settings highlight various data standardization/normalization scenarios. When using microarray data to impute microarray data, the training data and validation/testing data (including both measured and unmeasured genes) are quantile normalized to the same distribution. When using RNA-seq data to impute RNA-seq data, samples only undergo within-sample normalization (using TPM) without any between-sample normalization. When using RNA-seq data to impute microarray, the training set data is not jointly normalized but the validation/testing data are. The fact that microarray data has a much lower imputation error than the RNA-seq also points to RNA-seq profiles having very different data distributions due to not being (quantile) normalized across samples, coming from many different sequencing platforms, and having a broader dynamic range than microarray data. While we have performed an analysis showing that jointly normalizing RNA-seq and microarray data improves imputation (Supplementary Figure S19), future work is required to examine the effect of data normalization and transformation and to develop strategies to perhaps transform data just based on measured genes and, upon imputation, recast the unmeasured genes into the original data space (see Section 2.3 in the Supplemental Material for a further discussion on this topic).

The performance of the various imputation methods in the cross-technology imputation task, where the influence of data transformation is most evident, highlights how each method works to impute gene expression. *SampleKNN* performs poorly because, for a given microarray sample to impute, it finds the closest RNA-seq samples, which come from a different data distribution. On the other hand, *GeneKNN* has relatively good performance because it works completely within the training data (RNA-seq) to find the genes nearest to a particular gene and then uses these gene relationships for imputation within the microarray data. Even though *GeneLASSO* similarly captures gene relationships only using the training data (RNA-seq), the mapping in the form of model coefficients does not transfer to microarray samples as easily as nearest neighbors. Similar issues, in addition to potential overfitting to the training set, thwart the performance of *GeneDNN* (Supplementary Figure S28), although the additional complexity of *GeneGAN* helps deep neural networks to better transfer information from one technology to the other. *SampleLASSO*’s top performance in this setting stems from having the unique property of learning a supervised model that, in addition to learning meaningful sample relationships, naturally captures the scaling factors required to closely map the RNA-seq data distribution to the microarray data distribution.

Our results indicate that the deep learning models show good performance for imputation in the LINCS subset using microarray data to impute microarray data. This is to be expected as LINCS is the application for which these methods were optimized. The deep learning methods additionally show superior performance on the task of using RNA-seq data to impute RNA-seq data. This could be due to having more training examples for this task compared to the microarray to microarray task. The deep-learning methods further benefit from minimal variation in the RNA-seq data across platforms due to the uniform processing of the samples in the ARCHS4 database.

Nevertheless, deep-learning-based imputation techniques have a number of practical drawbacks. Foremost among them is the very large number of hyperparameters, in addition to other aspects of model optimization such as regularization and dropout, that need to be tuned to build an accurate model. This drawback is further amplified by the fact that these optimal parameters are likely to change depending on the platform or technology in question. Deep learning models are also hard to scale in terms of model size, with the number of weights growing nonlinearly with increasing layer number and layer size. Additionally, the current state of the standard hardware is such that only ∼32GB of a model can fit into the memory of a GPU, and anything more requires the utilization of multiple GPUs. For example, this is why methods such as *SampleDNN* or *SampleGAN* could not be implemented, as in these cases the input layer would be comprised of >70 000 units (i.e. the number of units equals the number of samples in the training set), and even a neural network with just one hidden layer of 9000 units would be over double the size of a *GeneDNN* model with three hidden layers of 9000 units each in the LINCS gene subset setting.

The analysis using the SEEK data—which has not been carried out in any other study on imputation and which reflects the real world task of imputing data from many expression platforms—highlights a few more limitations of the deep learning methods. First, the time it takes to get imputed expression values from deep learning methods is determined by the size of the training set, and once the model is trained, predicting test set values is relatively fast. Therefore, there was no difference in runtime for the deep learning models whether we imputed 10 000 samples or just 90, and this runtime ended up being >20 h for *GeneDNN* and >40 h for *GeneGAN* per platform using expensive GPU hardware. In contrast, *SampleLASSO* took just 4 h per platform in SEEK using only one processor on one computational node. As each *SampleLASSO* model is independent of the others, this is an ‘embarrassingly parallel’ task and can be greatly sped up by running on many CPUs at once.

Secondly, in the SEEK data analysis, the deep learning methods perform noticeably poorly for almost all platforms. This is likely due to the fact that, since we are only imputing 90 genes, deep learning methods no longer benefit from the multi-task learning that happens when many thousands of genes are all predicted at once. In contrast, *SampleLASSO* and *GeneLASSO* train a model for every sample or gene, respectively. This is an important real world consideration as a researcher only may wish to add a limited number of ‘genes of interest’ to a given set of microarray samples, and *SampleLASSO* allows for a much quicker exploratory analysis of combining datasets across platforms.

The performance of the deep learning methods on the SEEK data could be improved if the model hyperparameters were optimized for each platform. This is not realistic in practice as there are numerous ways of combining data from expression platforms, and the computational time and the technical expertise required to optimize a deep learning model in a given imputation setting are not available to most researchers. We also note that the deep learning methods perform best when the training and testing data are similar (i.e. analysis shown in Figures 2 and 4). However, the analysis using the SEEK data more closely mimics the situation of using RNA-seq data to impute microarray data where the training and testing data comes from different distributions. More broadly, cross-platform imputation is the most meaningful setting because imputing samples from a platform using training data of the same platform is not needed as both training and testing samples will measure the expression of the same set of genes. In this cross-platform setting, interesting future work could be in applying domain adaptation techniques to further decrease the imputation error.

*SampleLASSO* is a simple, intuitive, flexible model. The benefits of *SampleLASSO* emanate from the fact that a new machine learning model is trained on-the-fly for each new target sample that needs to be imputed based on the set of measured genes in that sample. Any set of genes can be measured/unmeasured in this setup, obviating the need for fixed pretrained models. While we acknowledge that there are many more deep learning architectures that could be applied to the unmeasured gene expression problem, we highlight the fact there also exists many ways to optimize simple and elegant methods like *SampleLASSO* by exploring different regularization schemes and shallow nonlinear algorithms, or by reducing the number of features using feature selection methods or dimensionality reduction techniques.

Finally, these benefits come along with *SampleLASSO*’s ability to leverage biological information specific to the new target sample, enabling easy interpretability. We specifically evaluated *SampleLASSO*’s interpretability on a large-scale using samples labeled to various tissues of origin. This analysis shows that, when imputing a sample from a specific tissue, the *SampleLASSO* model up-weights training samples from the same tissue in majority of the cases (Figure 6 and Supplementary Figure S27). The rest of the cases could be due to high tissue heterogeneity (as in blood) or factors other than tissue-type (e.g. disease status, drug dosage) being the dominant signal.

In conclusion, we propose *SampleLASSO*, a simple method for imputing the expression of unmeasured genes. Extensive evaluations and analyses demonstrate that *SampleLASSO* is accurate, flexible, and interpretable. We have made all the data and code from this study freely available on Zenodo and GitHub (https://github.com/krishnanlab/Expresto) to aid in reproducing all our findings. Using a convenient function in our code, researchers can also use *SampleLASSO* to readily impute unmeasured genes in their samples of interest in any of the following practical settings: (i) complete the expression profile of publicly-available microarray samples from any platform to make them comparable to the human whole-genome microarray, (ii) predict the expression of genes absent in standard microarrays (e.g. most non-protein coding genes) using RNA-seq to impute microarray samples and (iii) fill in and effectively use genome-scale chemical and genetic perturbation expression data from LINCS based on the measured landmark genes.

## DATA AVAILABILITY

The data from this study is available on Zenodo (https://doi.org/10.5281/zenodo.3971092) and all code is available on Github (https://github.com/krishnanlab/Expresto).

## Supplementary Material

gkaa881_Supplemental_File
