# Supplementary material for: A flexible, interpretable, and accurate approach for imputing the expression of unmeasured genes
Source: Nucleic Acids Res. 2020 Oct 19;48(21):e125. doi: 10.1093/nar/gkaa881 (PMC7708069; doi:10.1093/nar/gkaa881)
Supplement: gkaa881_Supplemental_File [file gkaa881_supplemental_file.pdf]

A Flexible, Interpretable, and Accurate Approach for Imputing the Expression of Unmeasured Genes - Supplemental Material

Section 1: Supplemental Material for Methods

Section 1.1: Pictorial Representation of Unmeasured Gene Imputation

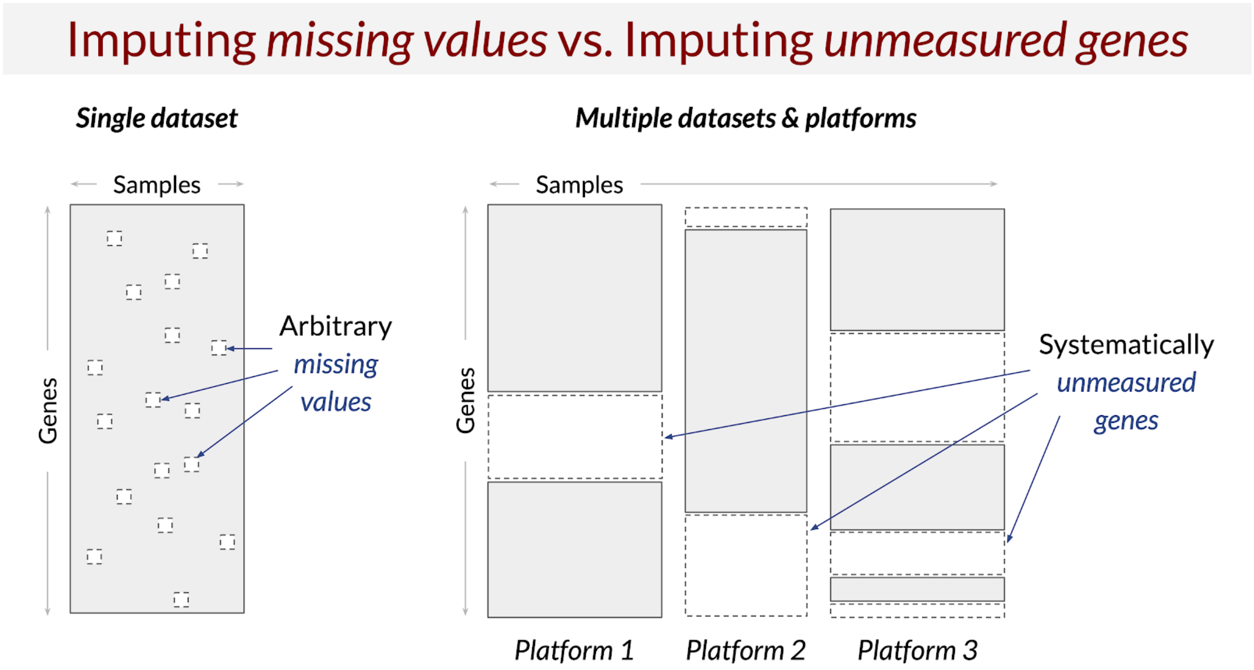

Fig. S1. Schematic of the difference between "missing value" imputation versus "unmeasured gene" imputation. In the missing value imputation problem, gene expression values from a single dataset that were unmeasured due to technical errors are imputed. In the unmeasured gene imputation problem, genes not measured at all by a given platform are imputed.

Section 1.2: Description of data processing steps

We downloaded the microarray data on Dec 6th 2017, and downloaded the RNA-seq data from the Sept 14th 2018 release of ARCHS4. In the microarray data, log transformation and quantile normalization was performed using Frozen Robust Multi-array Analysis (1) and the probes were mapped to Entrez space using a custom CDF (2). For the RNA-seq data, we converted the ENST IDs to Entrez IDs by taking the sum of all ENSTs mapped to a given ENSG, where the ENST to ENSG mapping was given by using the *gene2ensembl.gz* file available on the NCBI website on June 11th 2019. We note that the microarray data can be readily quantile normalized

as all the data comes from the *Affymetrix Human Genome U133 Plus 2.0 Array* platform. In contrast, quantile normalization of the RNA-seq data is not straightforward as the data was generated using many different sequencing platforms, and it is difficult to perform batch effect correction across this large number of expression samples. We mention that although there are 984 “landmark” genes in original LINCS data, the software package we used to map from microarray probes to Entrez gene IDs only contained 964 of the LINCS genes.

The procedure used to split the expression samples into the training, validation and test sets was; 1) samples were first grouped together if they appear in the same experiments, 2) a date is assigned to every one of these experiment groups by selecting the oldest date of an expression sample associated within the group, and 3) the experimental groups are then temporally split into training, validation and testing sets with the oldest experimental groups going into the training set and most recent experimental groups going into the test set. The split ratios were such that we had 80% of the data in the training, 10% of the data in the validation and testing sets. We then further subset the validation to 10% of it’s full size as described in the main text. We note, for the DNN and GAN methods we used all the validation data to most closely replicate the methods reported in (3, 4).

To get an idea if subsetting the validation data was sufficient we plotted the results for both the validation and test test for each method using the optimal hyperparameter [Fig. S2]. For both the Microarray-Microarray and RNAseq-Microarray cases, the performance between the two sets is nearly identical. For the situation where the validation and test data is RNA-seq (RNAseq-RNAseq), the test set performance is slightly worse than the validation set. However, we do not believe this is due to too few validation samples, rather it is due to the fact that since the data is split temporally, this is a batch effect as the sequencing technology is changing over time.

We downloaded the SEEK data (5) from http://seek.princeton.edu/download.jsp. We used all the platforms contained in this database except for *Affymetrix Human Genome U133 Plus 2.0 Array* and *Affymetrix Human Genome U133A Array* as this gene split was already considered in the first part of the manuscript. Detailed information about the SEEK data can be seen in Table S1.

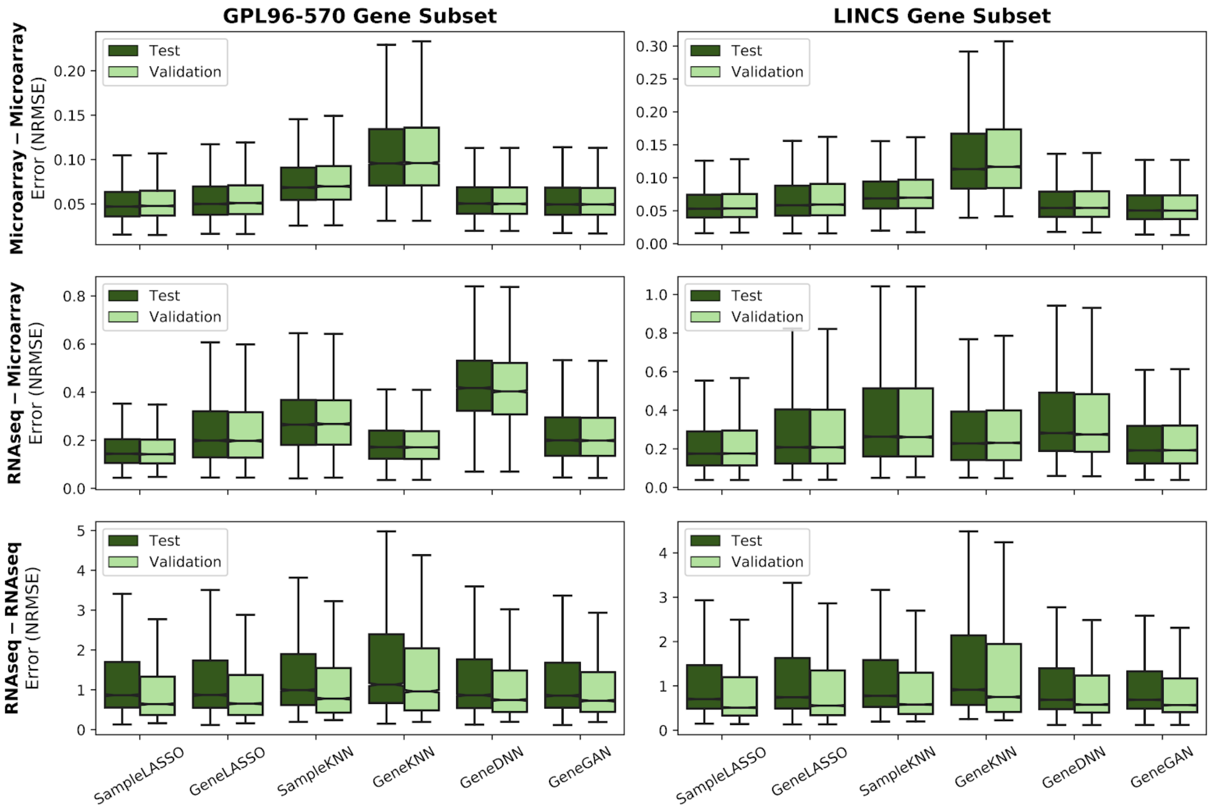

Fig. S2. Comparison of imputation methods across both validation and test sets. The performance of the methods is compared for the validation and test sets to see if using the subset validation set was sufficient. When the validation and testing data is microarray (top and middle rows) the performance is nearly identical. When the validation and test data is RNA-seq (bottom row) the test set performance is slightly worse than the validation set, but this effect is mostly likely due to the validation and test set containing expression samples from different sequencing platforms.

Table S1. Information on the SEEK Datasets

GPL (ID)	GPL (Name)	Total Number of Genes in GPL	Number of Samples for GPL in SEEK	Number of Measured Genes Used for Training
GPL5175	[HuEx-1_0-st] Affymetrix Human Exon 1.0 ST Array [transcript (gene) version]	19124	2407	16098
GPL6244	[HuGene-1_0-st] Affymetrix Human Gene 1.0 ST Array [transcript (gene) version]	19105	9384	16507
GPL571	[HG-U133A_2] Affymetrix Human Genome U133A 2.0 Array	11816	6782	11491
GPL8300	[HG_U95Av2] Affymetrix Human Genome U95 Version 2 Array	8188	2340	7878
GPL6480	Agilent-014850 Whole Human Genome Microarray 4x44K G4112F (Probe Name version)	18438	7815	16369
GPL4133	Agilent-014850 Whole Human Genome Microarray 4x44K G4112F (Feature Number version)	18249	6888	16243
GPL1708	Agilent-012391 Whole Human Genome Oligo Microarray G4112A (Feature Number version)	17311	2509	15798
GPL6884	Illumina HumanWG-6 v3.0 expression beadchip	18130	4679	16001
GPL6947	Illumina HumanHT-12 V3.0 expression beadchip	17053	6548	14772
TCGA	TCGA RNASeq V2 collection	15049	5085	13703

Section 1.3: Description of imputation methods

In this section, Figures S3-S7 provide a pictorial representation of how each imputation method was implemented. Following these figures is a description of all the parameters that were used for the *GeneDNN* and *GeneGAN* method. A schematic of the *GeneGAN* method can be seen in the original publication for that method (4).

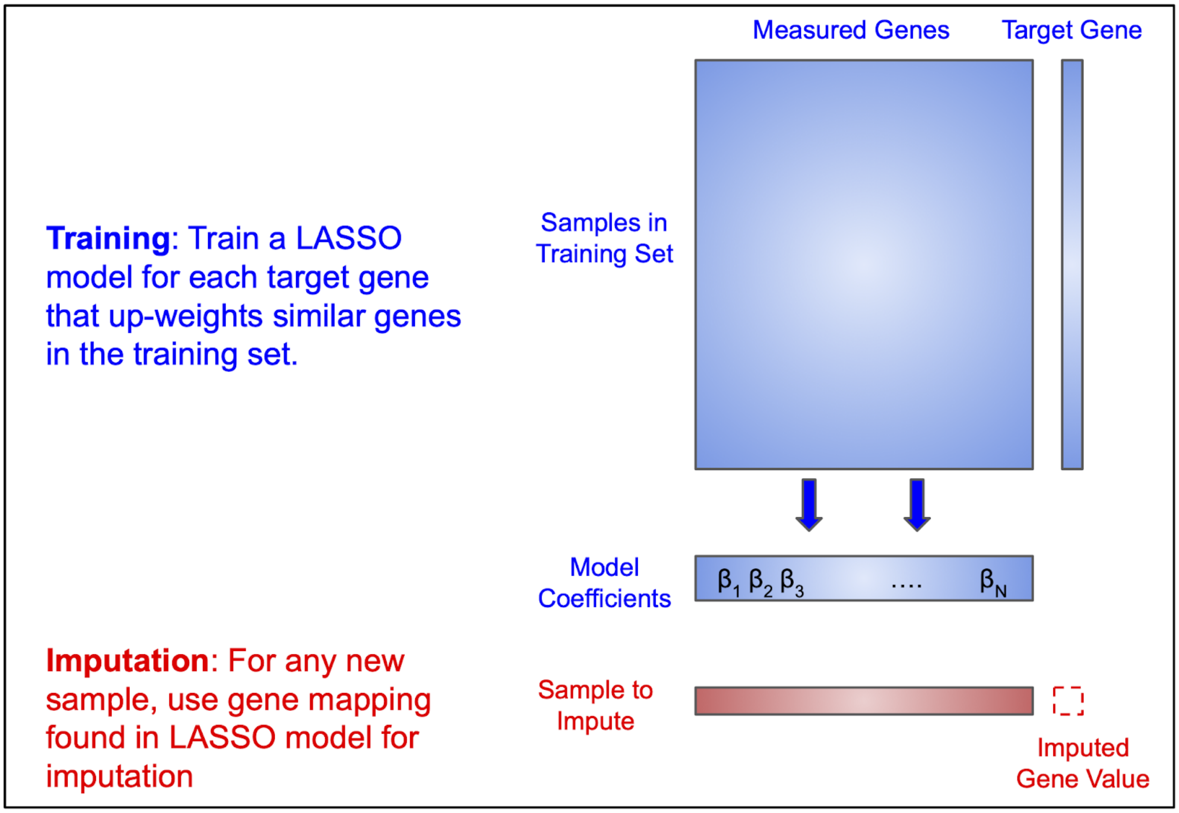

Fig. S3. Schematic of *GeneLASSO*. Blue color denotes training/fitting step and red color denotes imputation step.

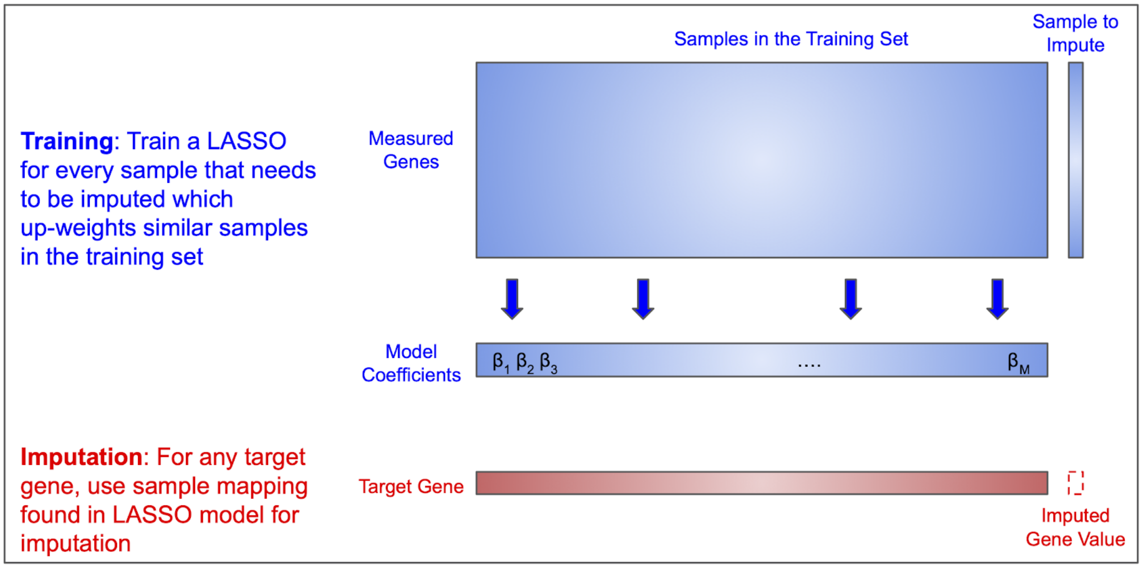

Fig. S4. Schematic of *SampleLASSO*. Blue color denotes training/fitting step and red color denotes imputation step.

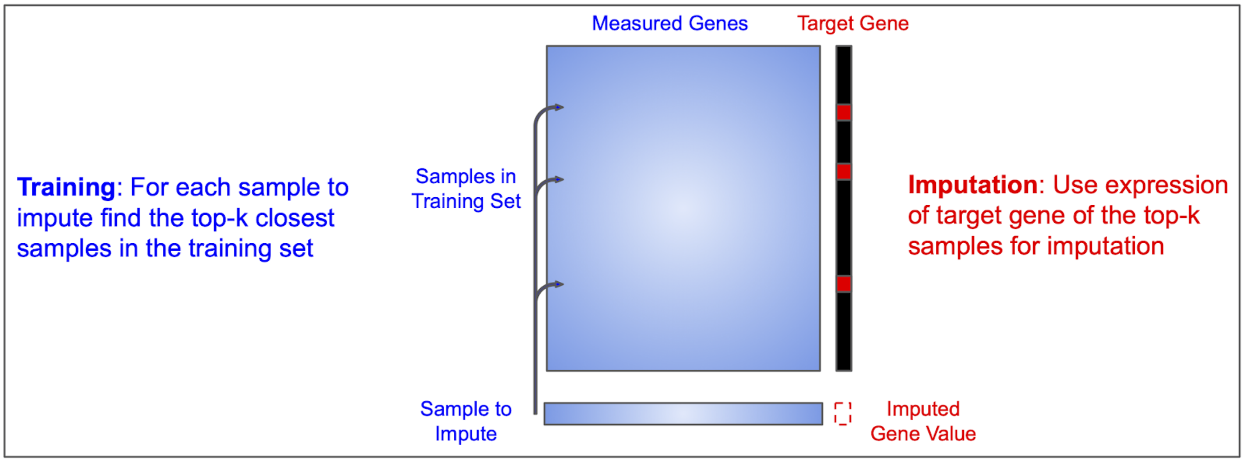

Fig. S5. Schematic of *SampleKNN*. Blue color denotes training/fitting step and red color denotes imputation step.

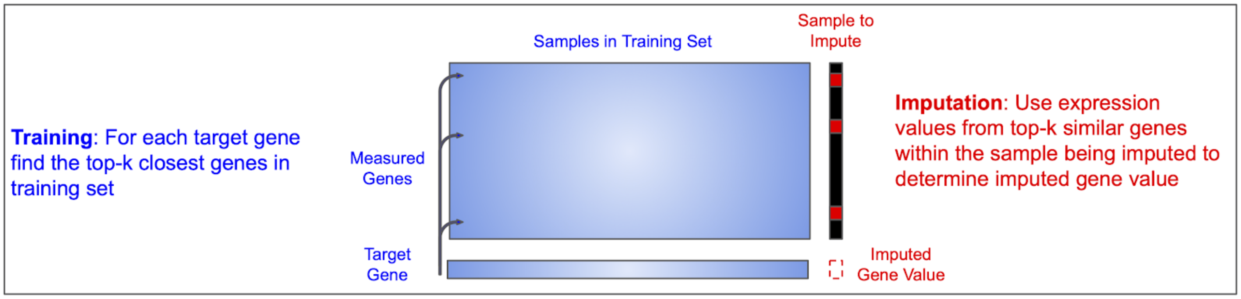

Fig. S6. Schematic of *GeneKNN*. Blue color denotes training/fitting step and red color denotes imputation step.

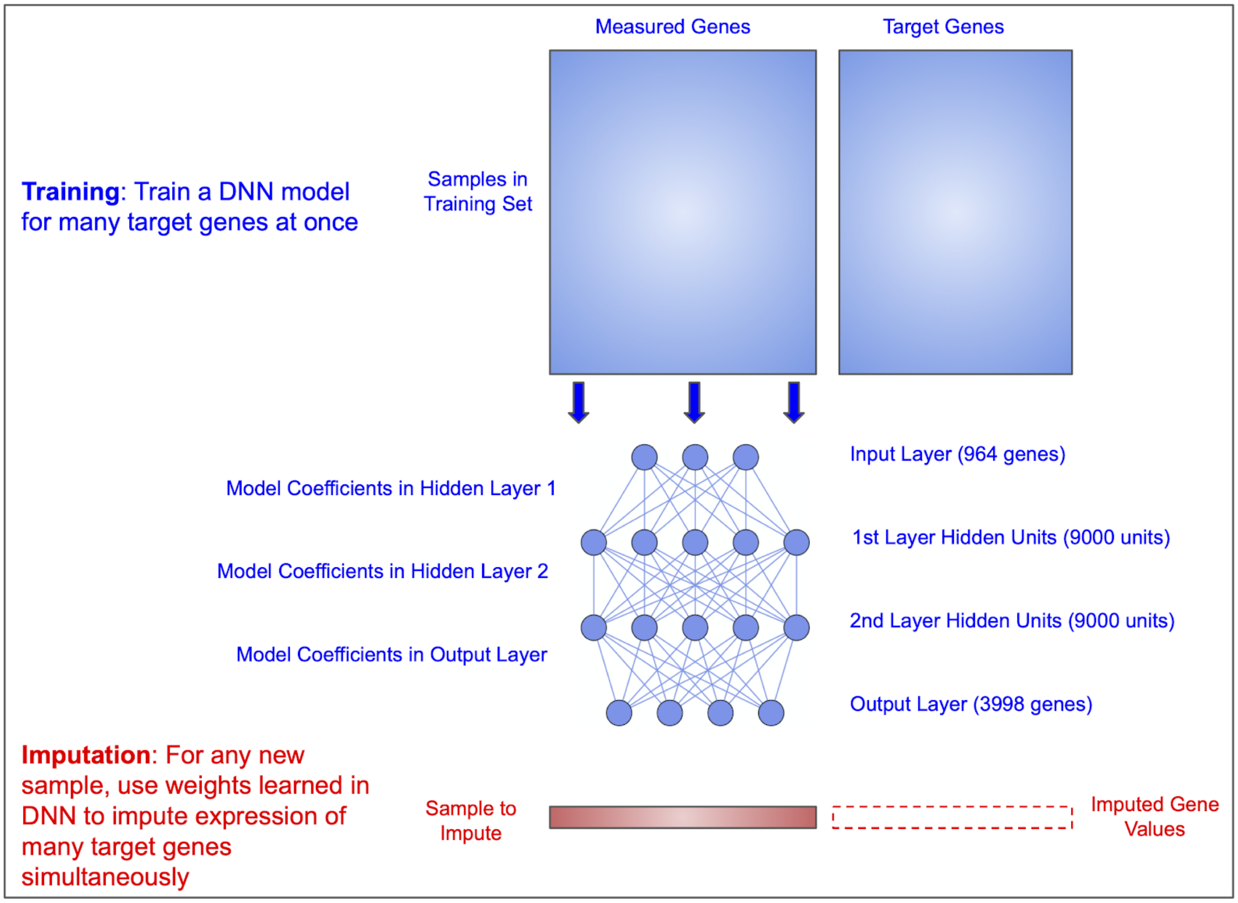

Fig. S7. Schematic of *GeneDNN*. Blue color denotes training/fitting step and red color denotes imputation step. A schematic of the *GeneGAN* method can be seen in Wang et al., 2018.

Parameters for the DNN

The model parameters for the *DNN* used in this work were chosen based on those used by *D-GEX* (3). This includes using a dropout rate of 10%, Xavier Uniform weight initialization (6), a mini-batch size of 200 and 200 training epochs. We chose 2 hidden layers with 9000 units in each layer as this architecture was the best overall for doing same-technology and cross-technology imputation. It was not obvious what exact optimizer was used by (3), so we performed hyperparameter tuning of the optimizer, using Adam (7, 8) and Adadelta (9). We implemented our *DNN* in *Keras* (10) using a *Tensorflow* backend (11).

In the original *D-GEX* paper, due to memory constraints, the target (unmeasured) genes were split into 4 sets, a separate *DNN* was trained for each set, and then the predictions were combined at the end. We implemented both settings: splitting into 4 sets and training 4 models (referred to here as *D-GEX*) as well trained a single *DNN* using all the target (unmeasured) genes in one model (referred to as *GeneDNN*). For all results presented in this work, we use the *GeneDNN* method as: 1) the performance of *D-GEX* and *GeneDNN* are nearly identical [Fig. S8] and 2) using all genes in the target set is how the *GAN* method is implemented. Further, our setup is consistent with growing memory efficiency due to which future implementations of *DNN*s are likely to include as many target genes in one model to increase the performance gained from transfer learning.

Parameters for the GAN

The model parameters for the *GAN* used in this work were chosen based on those used by *GGAN* (4). For the generator, we used a DenseNet architecture with 2 hidden layers with 9000 units each. We note that, in the *GGAN* paper, a three-hidden-layer model performed the best. However, this model would not fit into the memory of our GPUs. For the discriminator, we used a one-hidden-layer network with 3000 hidden units. The loss functions, weight normalization, layer normalization, and hidden layer activation functions are all implemented based on the description in the *GGAN* paper. We did not implement an exponential decay learning rate with Adam as they did in the *GGAN* paper, although one optimizer we did try was Adadelta, which has an adaptive learning rate. We trained each model for 200 epochs or for a maximum time of 48 hours, whichever came first. Since there was no code released with the *GGAN* model, we had to build it from scratch ourselves, and therefore, some differences between our implementation and the original implementation might exist.

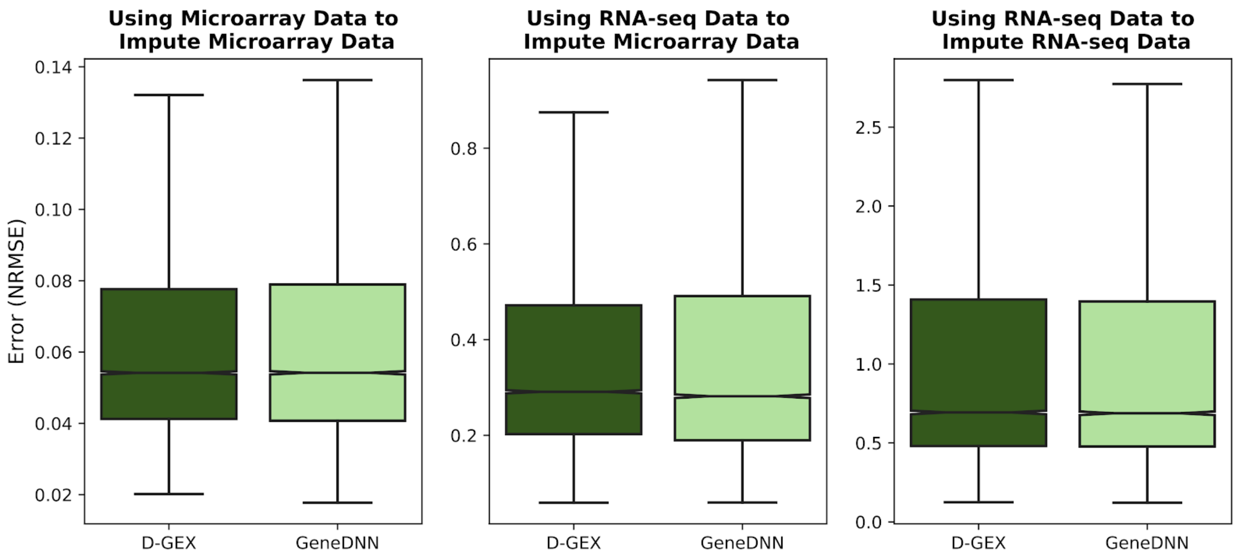

Fig. S8. Performance comparison between *GeneDNN* and *D-GEX*. The performance between *GeneDNN* and *D-GEX* on the LINCS gene split is nearly identical.

Section 1.4: Hyperparameter Tuning

Hyperparameter tuning was performed for all combinations of methods (*SampleLASSO*, *GeneLASSO*, *SampleKNN*, *GeneKNN*, *GeneDNN*, *GeneGAN*, and *D-GEX*), gene subsets (GPL96-570, LINCS) and tasks (Microarray-Microarray, RNAseq-Microarray, RNAseq-RNAseq). In the *LASSO* methods, the hyperparameter that was tuned was the strength of the L1-regularization, referred to as alpha. In the *KNN* methods, the hyperparameter that was tuned was the number of closest samples, referred to a k. In the deep learning methods, the hyperparameter that was tuned was the optimizer and the learning rate (Adadelta does not require a learning rate to be set), and the results show the error using the full validation set after the best model was selected across all epochs. All results are shown for NRMSE. The results of the hyperparameter tuning can be seen in Figs. S9-S15. Table S2 shows the hyperparameter that yielded optimal performance.

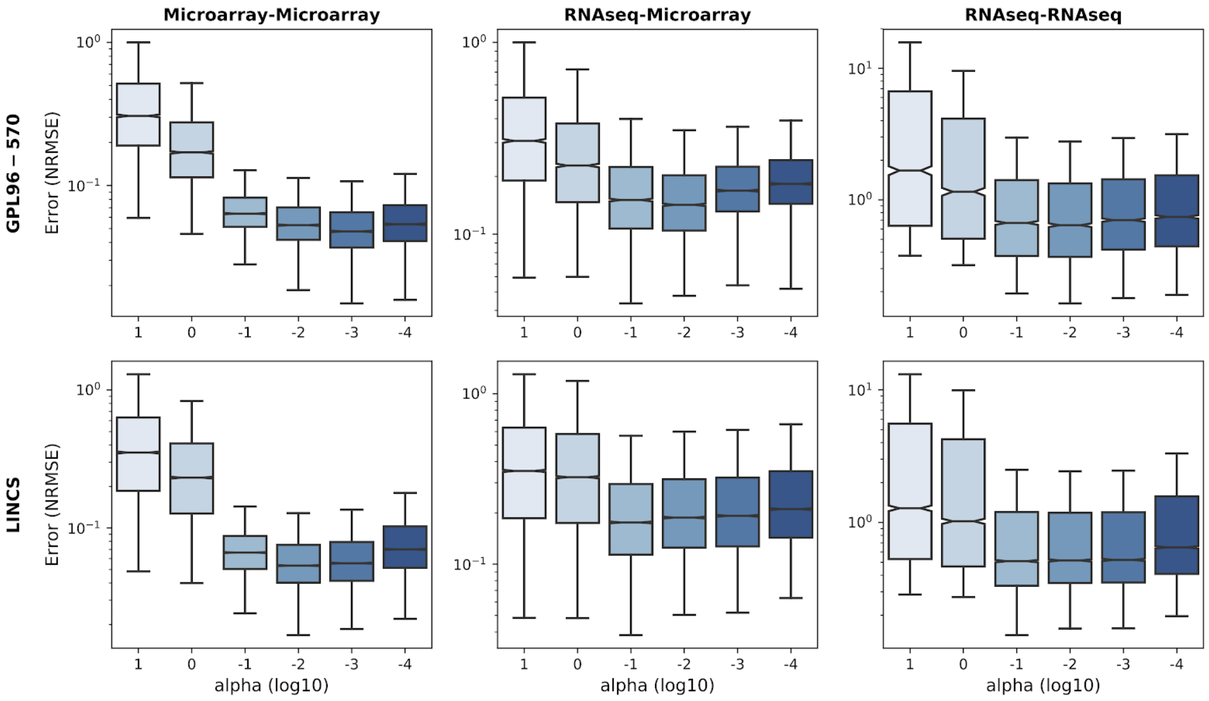

Fig. S9. Hyperparameter tuning for *SampleLASSO*.

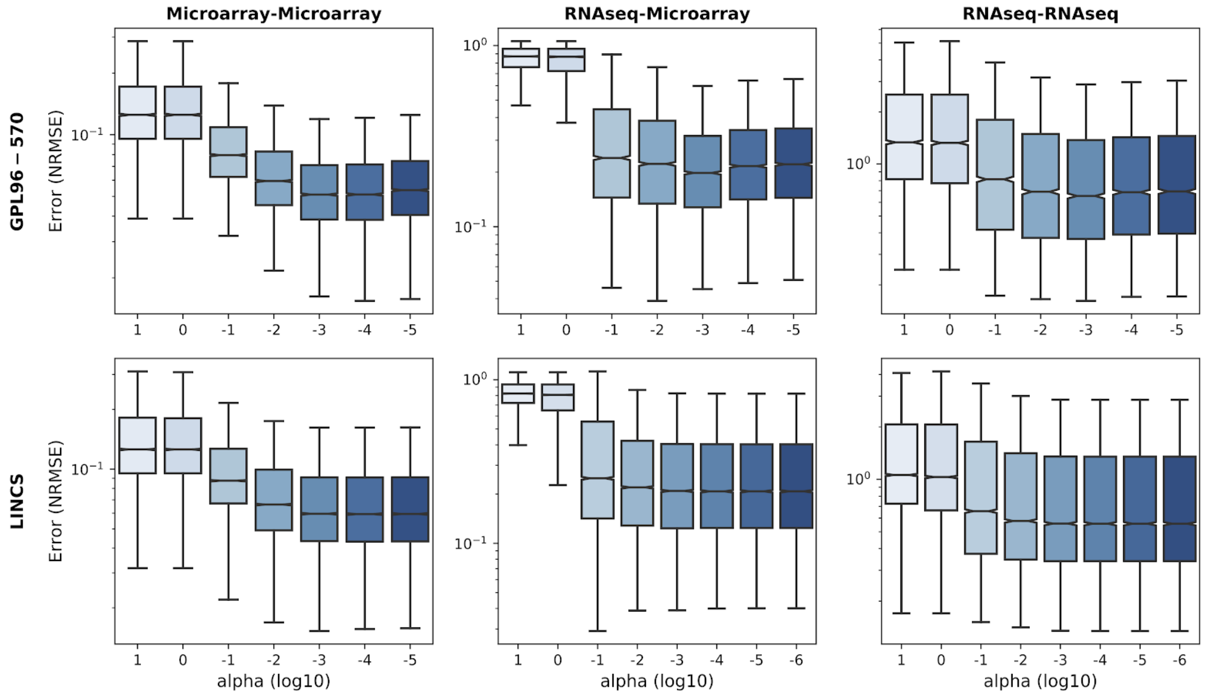

Fig. S10. Hyperparameter tuning for *GeneLASSO*.

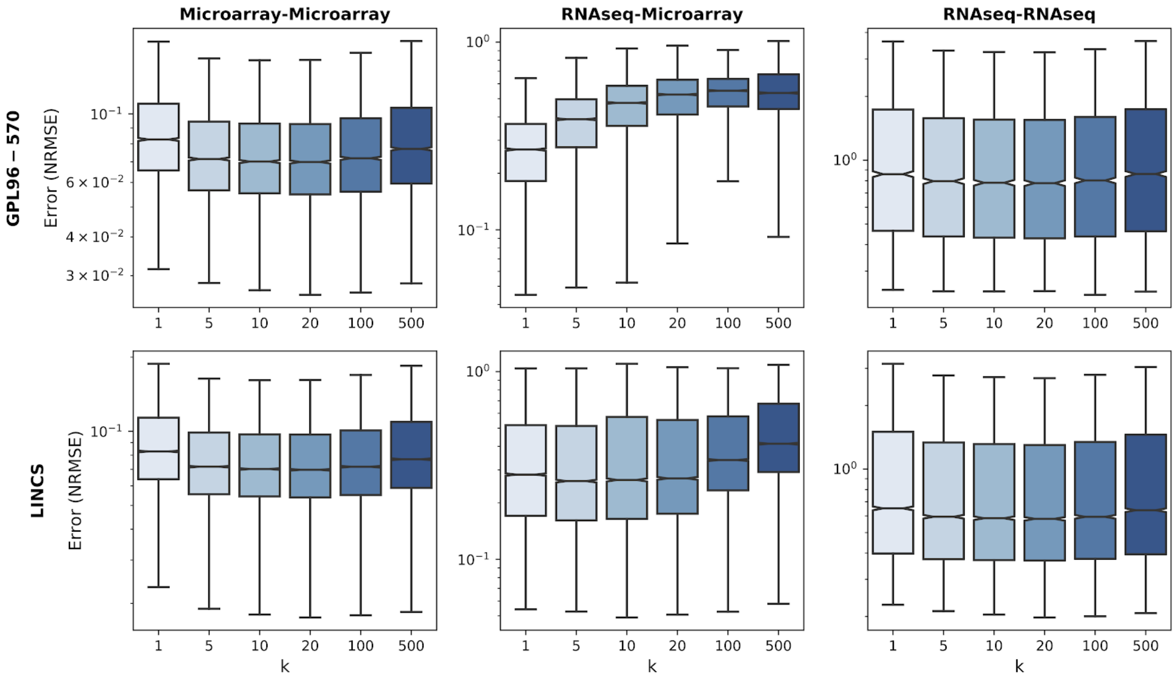

Fig. S11. Hyperparameter tuning for *SampleKNN*.

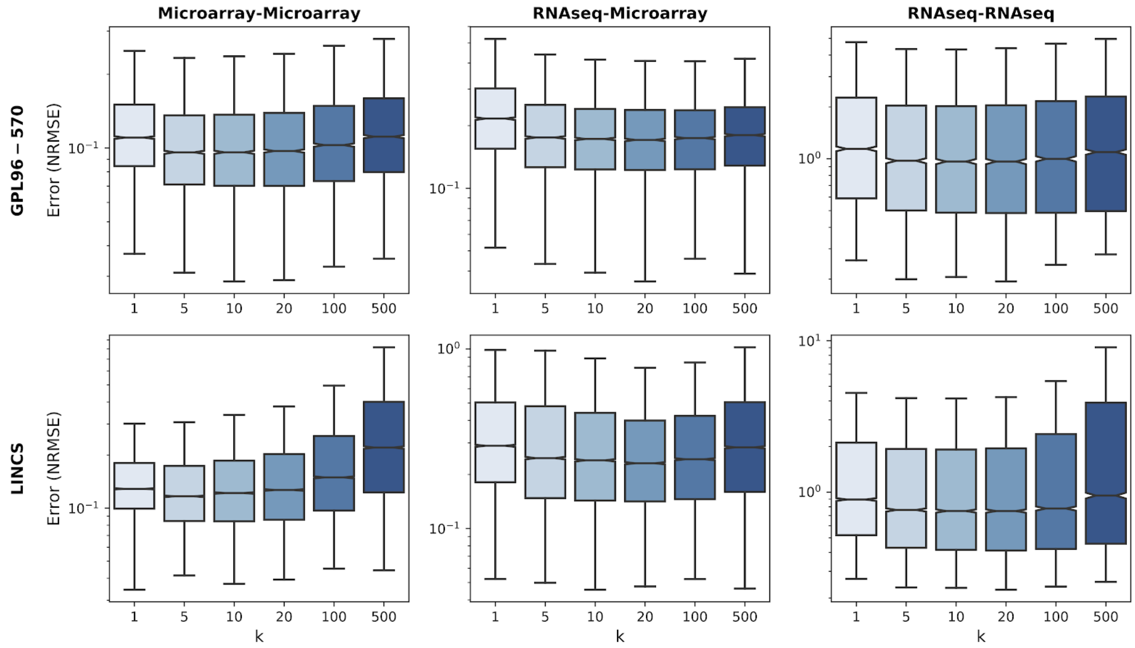

Fig. S12. Hyperparameter tuning for *GeneKNN*.

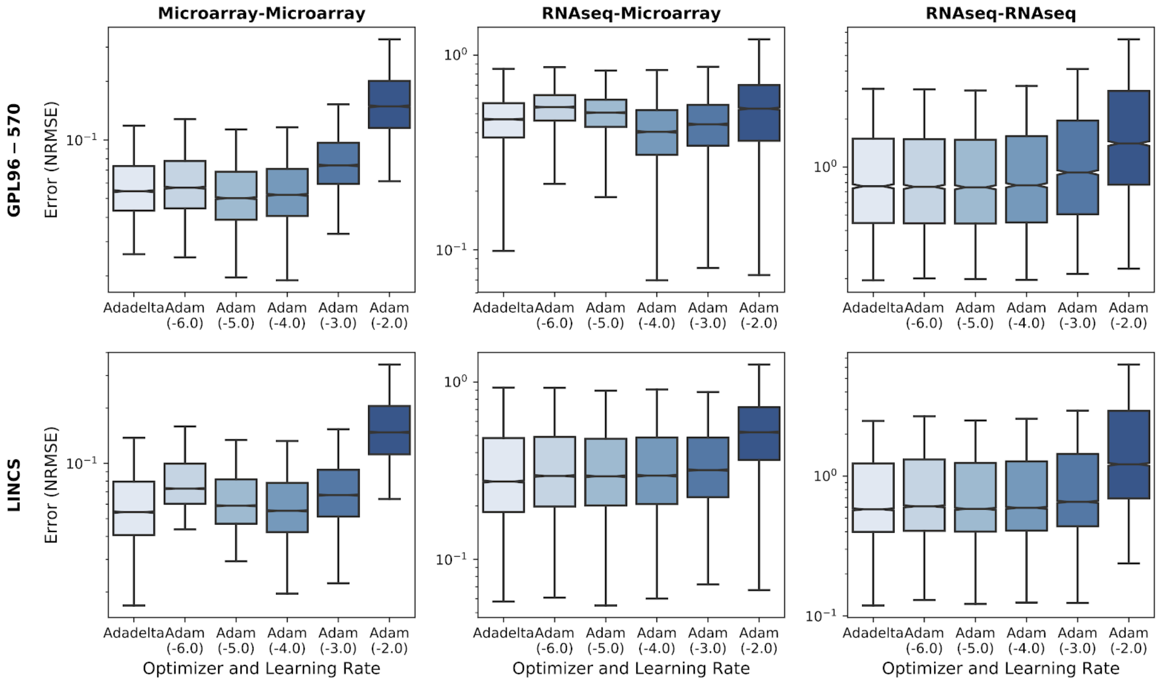

Fig. S13. Hyperparameter tuning for *GeneDNN*.

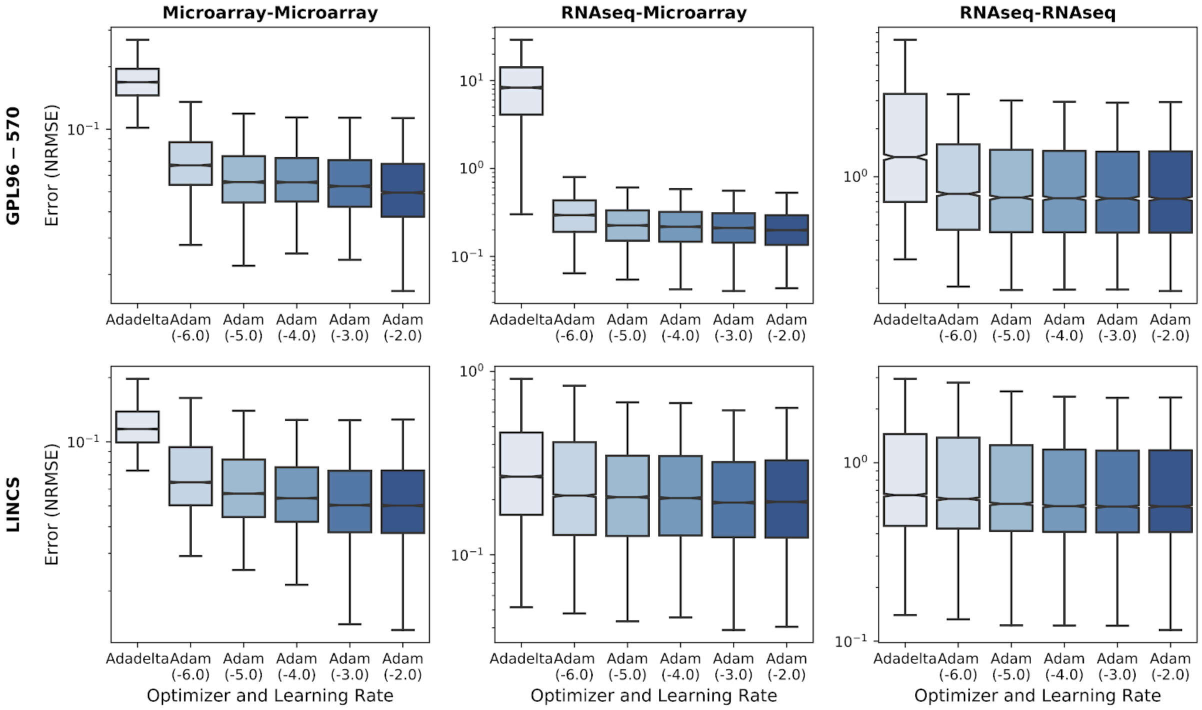

Fig. S14. Hyperparameter tuning for *GeneGAN*.

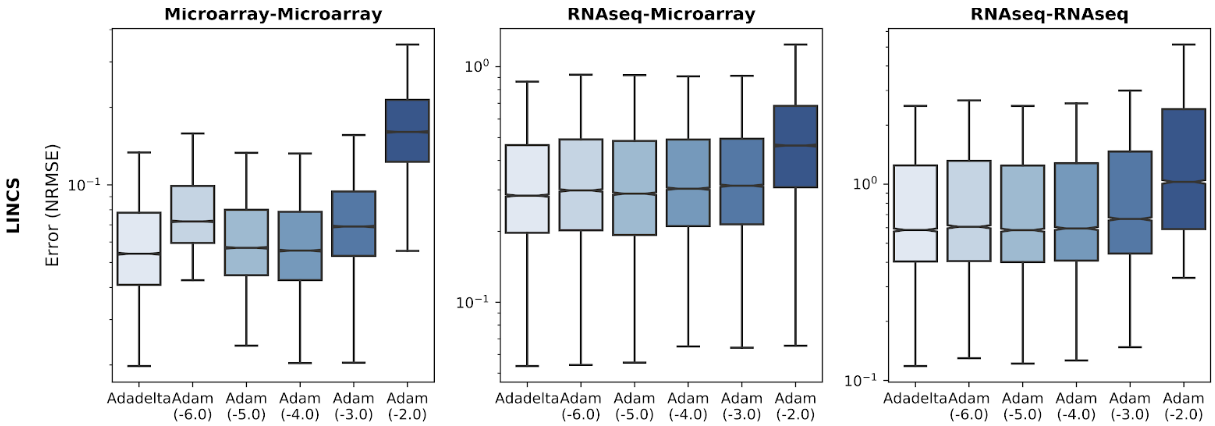

Fig. S15. Hyperparameter tuning for *D-GEX*.

Table S2: Best Models Settings from Hyperparameter Tuning. The NRMSE value is the median NRMSE value across all genes for that hyperparameter set.

		Method	Model Setting	Optimal Choice	NRMSE
Microarray-Microarray	GPL96-570	GeneDNN	optimizer	Adam (1e-05)	0.0502
	GPL96-570	GeneGAN	optimizer	Adam (0.01)	0.0495
	GPL96-570	GeneKNN	k	5.0	0.0961
	GPL96-570	GeneLASSO	alpha	0.001	0.0510
	GPL96-570	SampleKNN	k	20.0	0.0699
	GPL96-570	SampleLASSO	alpha	0.001	0.0478
	LINCS	D-GEX	optimizer	Adadelta	0.0540
	LINCS	GeneDNN	optimizer	Adadelta	0.0543
	LINCS	GeneGAN	optimizer	Adam (0.01)	0.0502
	LINCS	GeneKNN	k	5.0	0.1167
	LINCS	GeneLASSO	alpha	0.0001	0.0593
	LINCS	SampleKNN	k	20.0	0.0698
	LINCS	SampleLASSO	alpha	0.01	0.0534
RNAseq-Microarray	GPL96-570	GeneDNN	optimizer	Adam (0.0001)	0.4033
	GPL96-570	GeneGAN	optimizer	Adam (0.01)	0.1990
	GPL96-570	GeneKNN	k	20.0	0.1708
	GPL96-570	GeneLASSO	alpha	0.001	0.1980
	GPL96-570	SampleKNN	k	1.0	0.2677
	GPL96-570	SampleLASSO	alpha	0.01	0.1422
	LINCS	D-GEX	optimizer	Adadelta	0.2837
	LINCS	GeneDNN	optimizer	Adadelta	0.2748
	LINCS	GeneGAN	optimizer	Adam (0.001)	0.1927
	LINCS	GeneKNN	k	20.0	0.2309
	LINCS	GeneLASSO	alpha	0.0001	0.2078
	LINCS	SampleKNN	k	5.0	0.2612
	LINCS	SampleLASSO	alpha	0.1	0.1757
RNAseq-RNAseq	GPL96-570	GeneDNN	optimizer	Adam (1e-05)	0.7468
	GPL96-570	GeneGAN	optimizer	Adam (0.01)	0.7281
	GPL96-570	GeneKNN	k	20.0	0.9632
	GPL96-570	GeneLASSO	alpha	0.001	0.6533
	GPL96-570	SampleKNN	k	20.0	0.7792
	GPL96-570	SampleLASSO	alpha	0.01	0.6410
	LINCS	D-GEX	optimizer	Adam (1e-05)	0.5833
	LINCS	GeneDNN	optimizer	Adadelta	0.5788
	LINCS	GeneGAN	optimizer	Adam (0.001)	0.5681
	LINCS	GeneKNN	k	20.0	0.7506
	LINCS	GeneLASSO	alpha	1e-05	0.5560
	LINCS	SampleKNN	k	20.0	0.5811
	LINCS	SampleLASSO	alpha	0.1	0.5107

Table S3: Detailed information on comparing the methods. Median: the median NRMSE value across all genes for that method. Percent SL Better: the percentage of times *SampleLASSO* is better than the other method. Log2 Effect Size: the log2 increase of *SampleLASSO* over the other method considering just the median value (a positive value is when *SampleLASSO* is the better performing method). P-Value: the significance between *SampleLASSO* and the other method based on a Wilcoxon rank-sum test.

		Median	Percent SL Better	Log2 Effect Size	P-Value
**Microarray-Microarray-GPL96-570**	**SampleLASSO**	0.047	N/A	N/A	N/A
	**GeneGAN**	0.050	0.92	0.073	0.00e+00
	**GeneLASSO**	0.050	0.91	0.088	0.00e+00
	**GeneDNN**	0.050	0.95	0.101	0.00e+00
	**SampleKNN**	0.069	1.00	0.542	0.00e+00
	**GeneKNN**	0.096	1.00	1.024	0.00e+00
**Microarray-Microarray-LINCS**	**GeneGAN**	0.050	0.25	-0.073	0.00e+00
	**SampleLASSO**	0.053	N/A	N/A	N/A
	**GeneDNN**	0.054	0.77	0.032	0.00e+00
	**GeneLASSO**	0.058	0.91	0.137	0.00e+00
	**SampleKNN**	0.069	0.98	0.373	0.00e+00
	**GeneKNN**	0.113	1.00	1.094	0.00e+00
**RNAseq-Microarray-GPL96-570**	**SampleLASSO**	0.144	N/A	N/A	N/A
	**GeneKNN**	0.172	0.70	0.254	4.38e-223
	**GeneLASSO**	0.199	0.71	0.467	2.21e-299
	**GeneGAN**	0.200	0.71	0.474	2.13e-261
	**SampleKNN**	0.265	0.80	0.881	0.00e+00
	**GeneDNN**	0.417	0.91	1.536	0.00e+00
**RNAseq-Microarray-LINCS**	**SampleLASSO**	0.175	N/A	N/A	N/A
	**GeneGAN**	0.192	0.56	0.134	5.28e-91
	**GeneLASSO**	0.208	0.62	0.251	0.00e+00
	**GeneKNN**	0.228	0.84	0.384	0.00e+00
	**SampleKNN**	0.263	0.76	0.589	0.00e+00
	**GeneDNN**	0.282	0.76	0.688	0.00e+00
**RNAseq-RNAseq-GPL96-570**	**GeneGAN**	0.854	0.40	-0.019	2.90e-26
	**GeneDNN**	0.864	0.39	-0.002	3.93e-02
	**SampleLASSO**	0.866	N/A	N/A	N/A
	**GeneLASSO**	0.871	0.41	0.009	6.96e-03
	**SampleKNN**	0.993	0.93	0.198	0.00e+00
	**GeneKNN**	1.133	0.98	0.388	0.00e+00
**RNAseq-RNAseq-LINCS**	**GeneDNN**	0.688	0.13	-0.032	0.00e+00
	**GeneGAN**	0.688	0.31	-0.032	0.00e+00
	**SampleLASSO**	0.703	N/A	N/A	N/A
	**GeneLASSO**	0.744	0.62	0.082	0.00e+00
	**SampleKNN**	0.777	0.84	0.145	0.00e+00
	**GeneKNN**	0.914	1.00	0.379	0.00e+00

Section 2: Supplemental Results

Section 2.1: Detailed Information on Method Comparisons

Table S3 shows detailed information between the comparison of all the methods.

Section 2.2: Results for Spearman and MAE metrics

In this section, we present the results in terms of the spearman correlation and the mean absolute error (MAE). The spearman correlation for a gene, (g_i), is given by

$$Spearman(g_i) = \frac{cov(\hat{x}_R, x_R)}{\sigma_{\hat{x}_R}\sigma_{x_R}} \qquad \text{eqn. S1}$$

where $\hat{x}_R$ and x_R are the imputed and real values for all the samples converted to ranks, respectively, cov is the covariance and σ is the standard deviation. The mean absolute error for a gene, (g_i), is given by

$$MAE(g_i) = \sum_{j=1}^{S} \left| \hat{g}_{i,j} - g_{i,j} \right| /S \qquad \text{eqn. S2}$$

Where S is the number of samples, and $\hat{g}_{i,j}$, $g_{i,j}$ are the imputed and real expression values, respectively, for the ith gene in the jth sample.

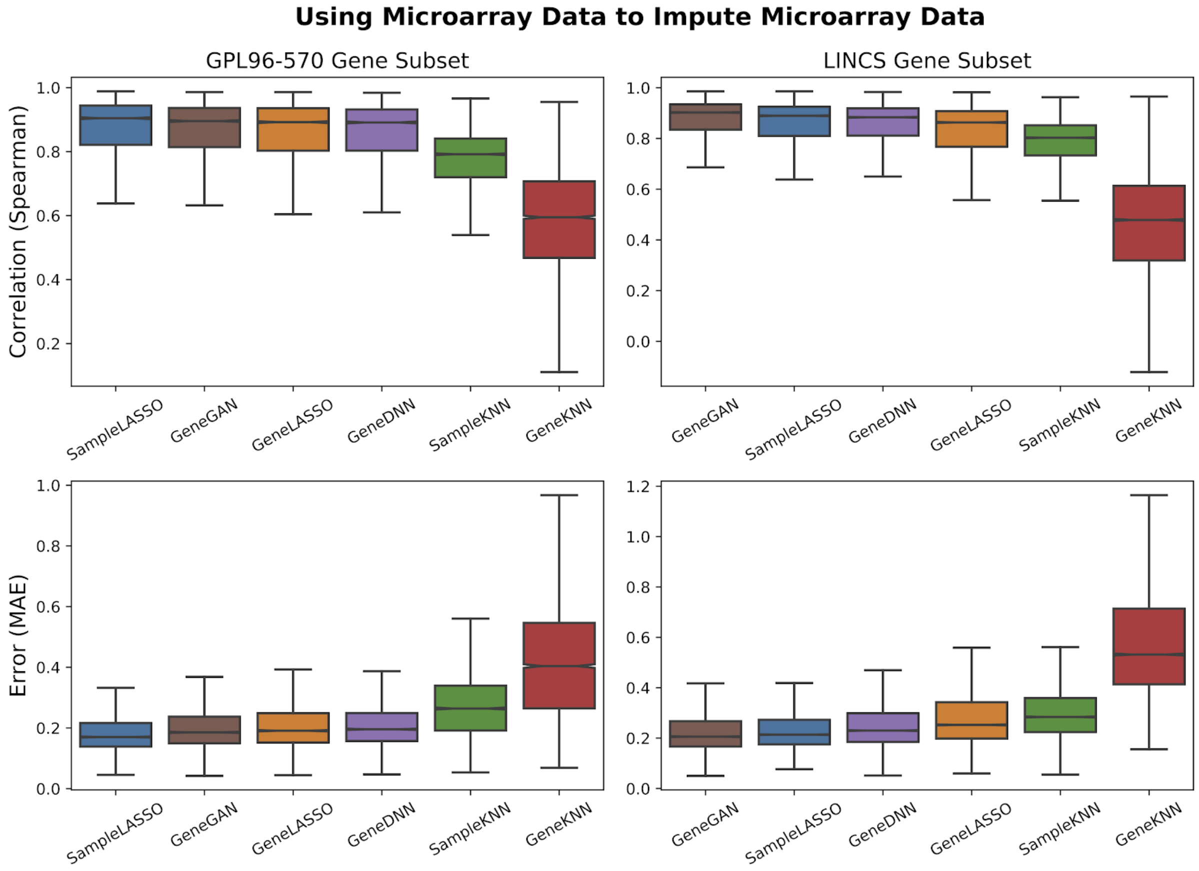

Fig S16. Performance of imputation models on microarray data with Spearman and MAE metrics. The performance of the six methods imputation models (*SampleLASSO*, *GeneDNN*, *GeneLASSO*, *SampleKNN*, *GeneKNN*, *GeneGAN*) are compared for using microarray data to impute microarray data.

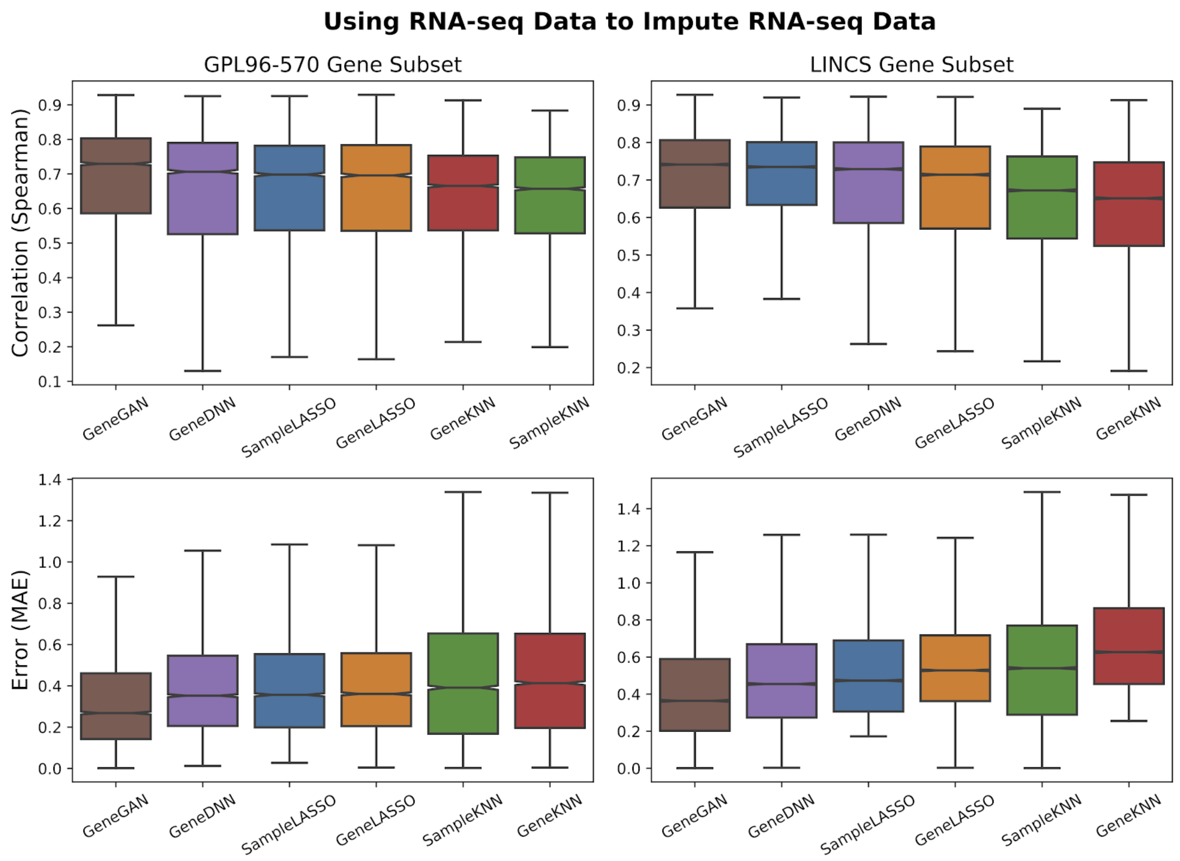

Fig S17. Performance of imputation models on RNA-seq data with Spearman and MAE metrics. The performance of the six methods imputation models (*SampleLASSO*, *GeneDNN*, *GeneLASSO*, *SampleKNN*, *GeneKNN*, *GeneGAN*)) are compared for using RNA-seq data to impute RNA-seq data.

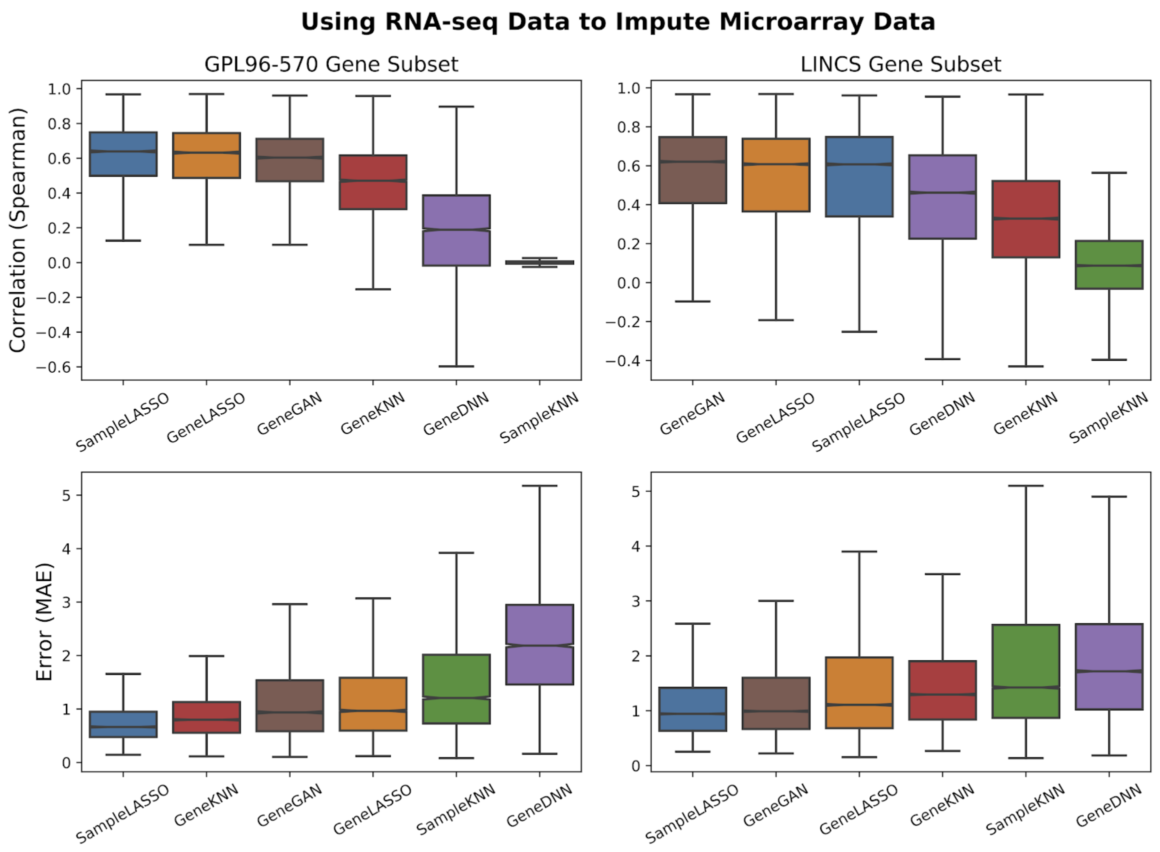

Fig S18. Performance of imputation methods for cross-technology imputation with Spearman and MAE metrics. The performance of the six methods imputation models (*SampleLASSO*, *GeneDNN*, *GeneLASSO*, *SampleKNN*, *GeneKNN*, *GeneGAN*) are compared for using RNA-seq data to impute microarray data.

Section 2.3: Effects of normalization on imputation

To assess how applying a basic normalization technique can improve imputation accuracy, we jointly normalized the data used for the task of using RNA-seq data to impute microarray data. For this analysis, we first quantile normalized the microarray test set data and then transformed the RNA-seq training data to this space. We then compared using this normalization method to only considering quantile normalizing the microarray test set data. It was necessary to perform the quantile normalization on the microarray test set data in the “No Normalization” setting to allow for the imputed values to be equivalent between both imputation settings. The imputation was done for 1000 random samples in the test set for the LINCS gene subset. We see that performing a joint quantile normalization between the RNA-seq and microarray data increases the performance substantially [Fig. S19]. We note that even for the “No Normalization” case, the errors are higher than those observed in Fig. 3. This could be due to the fact the test data used in this analysis and that used in the main manuscript are on different scales due to the quantile normalization being performed on the test set in this analysis.

Although this type of joint normalization has been done in all recent imputation studies involving data from different platforms and technologies, we note that this normalization procedure cannot be applied to real-world imputation in a straightforward manner. The reason for this is that, in a real imputation case, the unmeasured genes in the samples to be imputed are actually not known. Whereas in an evaluation study, we create mock ‘unmeasured genes’ and use their known ground truth values of the unmeasured genes to evaluate the imputation methods. In addition to being able incorporate this distribution of expression values from the unmeasured genes into normalization methods, an interesting avenue of future exploration will be in designing normalization methods that could either transform the samples to be imputed into the space of the training set samples, or vice versa.

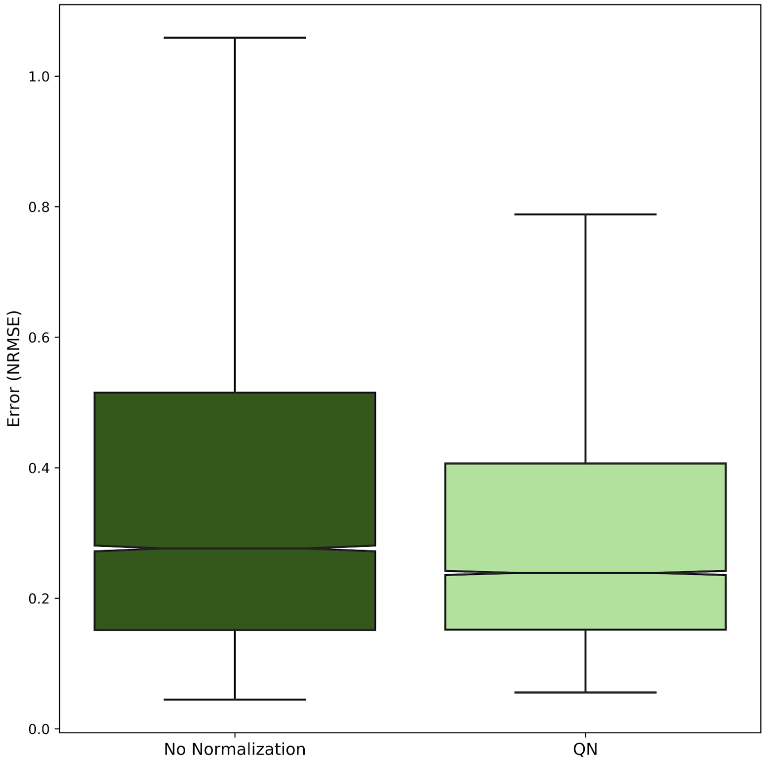

Fig S19. Performance comparison for using normalization and no normalization. The task is using RNA-seq data to predict microarray data and the gene subset is LINCS. QN (light green) refers to the case of performing joint quantile normalization across the RNA-seq and microarray datasets.

Section 2.4: Evaluations considering the expression levels and variance

In this section, we analyze how the performance of the methods changes for two gene properties; the mean expression of a gene and the variance of the expression. Genes were split up into low, medium and high bins for each property, and each panel in a figure [Figs. S20-S25] is the intersection of genes included in the two bins. See the plotting notebook in the associated

GitHub repo (https://github.com/krishnanlab/Expresto) for the breakdown of mean and variance values in each bin as well as the number of points in each boxplot.

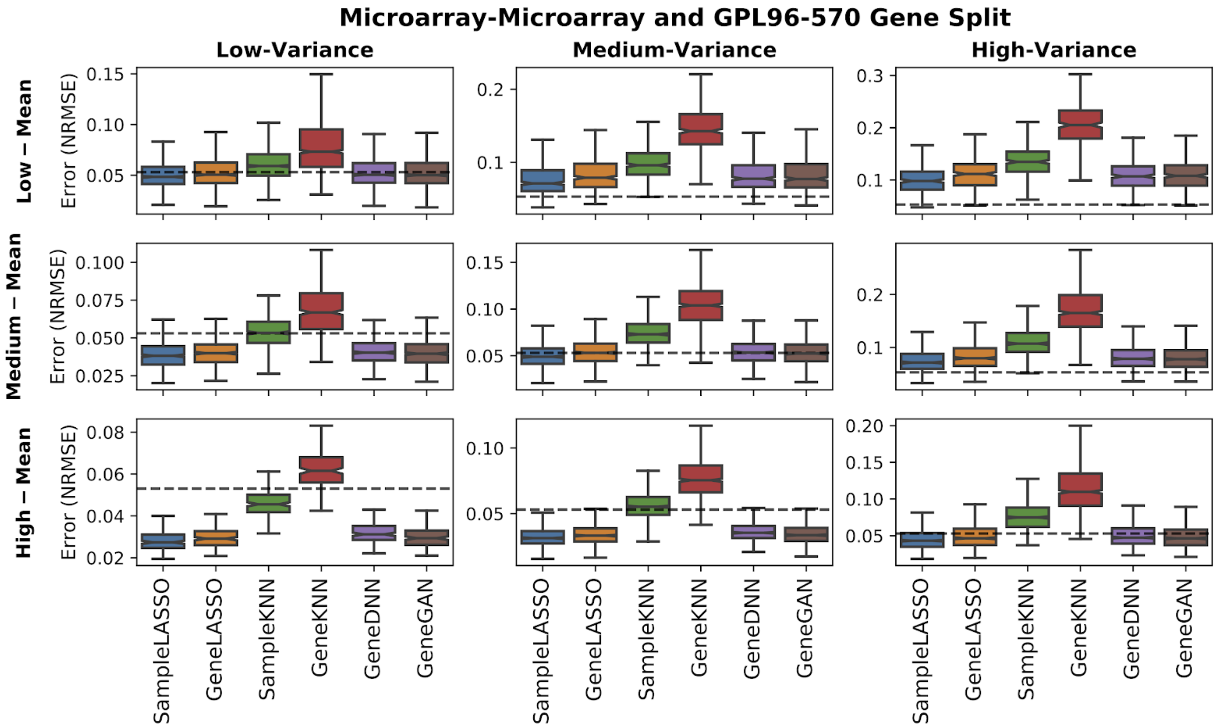

Fig. S20. Results broken up by mean and variance of gene expression for using microarray data to impute microarray data for the GPL96-570 gene subset. The dotted line is the median value when considering all genes for *SampleLASSO* (this is to help compare performances across the panels).

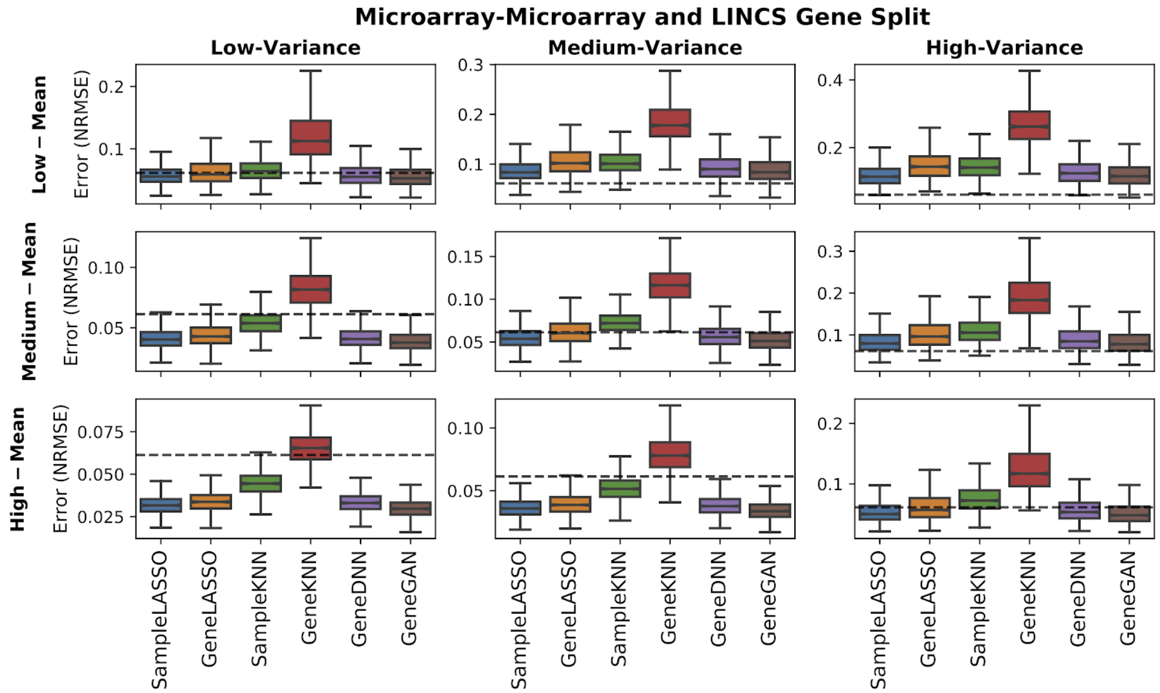

Fig. S21. Results broken up by mean and variance of gene expression for using microarray data to impute microarray data for the LINCS gene subset. The dotted line is the median value when considering all genes for *SampleLASSO* (this is to help compare performances across the panels).

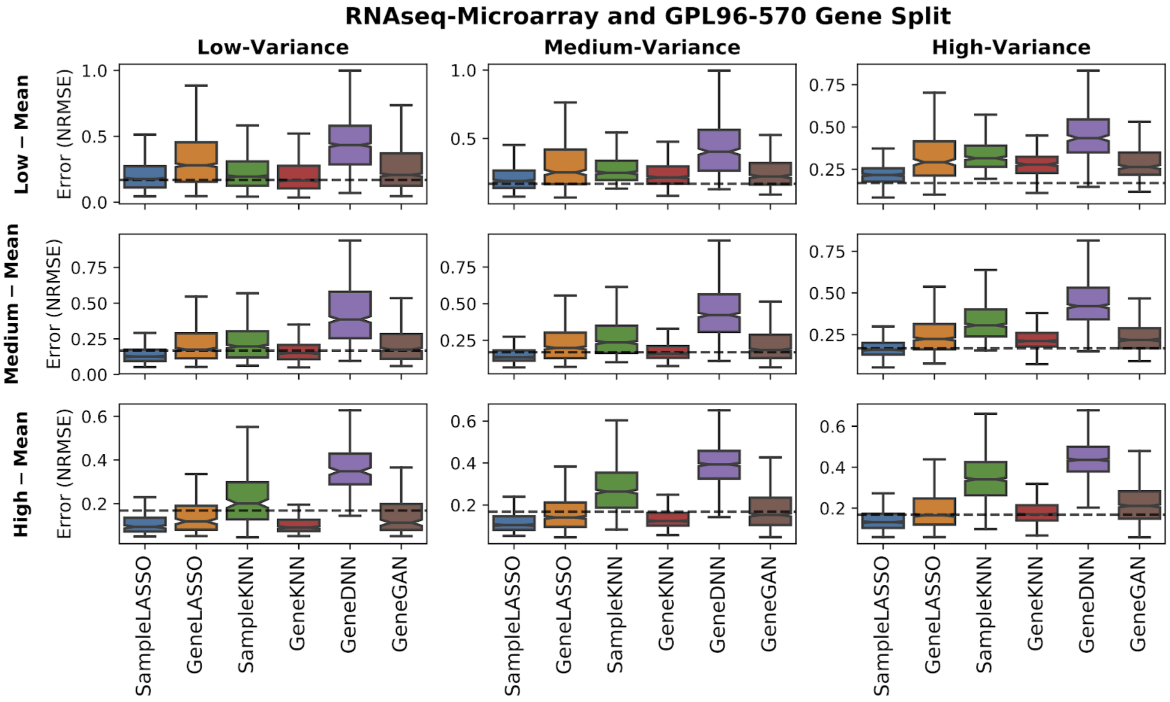

Fig. S22. Results broken up by mean and variance of gene expression for using RNA-seq data to impute microarray data for the GPL96-570 gene subset. The dotted line is the median value when considering all genes for *SampleLASSO* (this is to help compare performances across the panels).

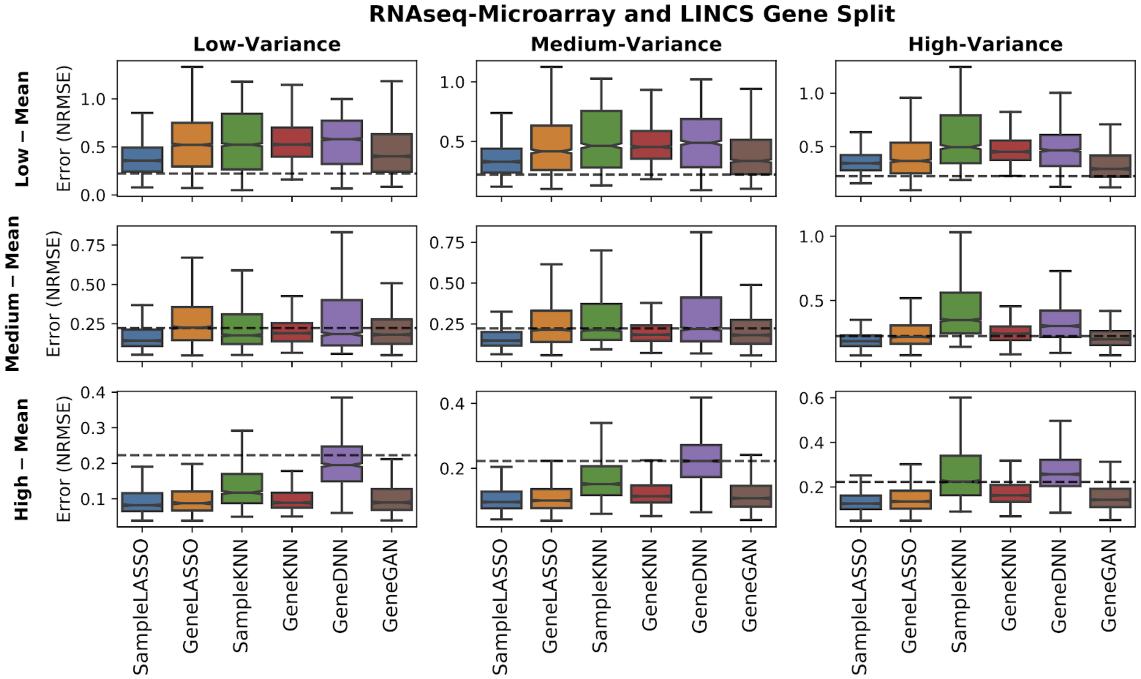

Fig. S23. Results broken up by mean and variance of gene expression for using RNA-seq data to impute microarray data for the LINCS gene subset. The dotted line is the median value when considering all genes for *SampleLASSO* (this is to help compare performances across the panels).

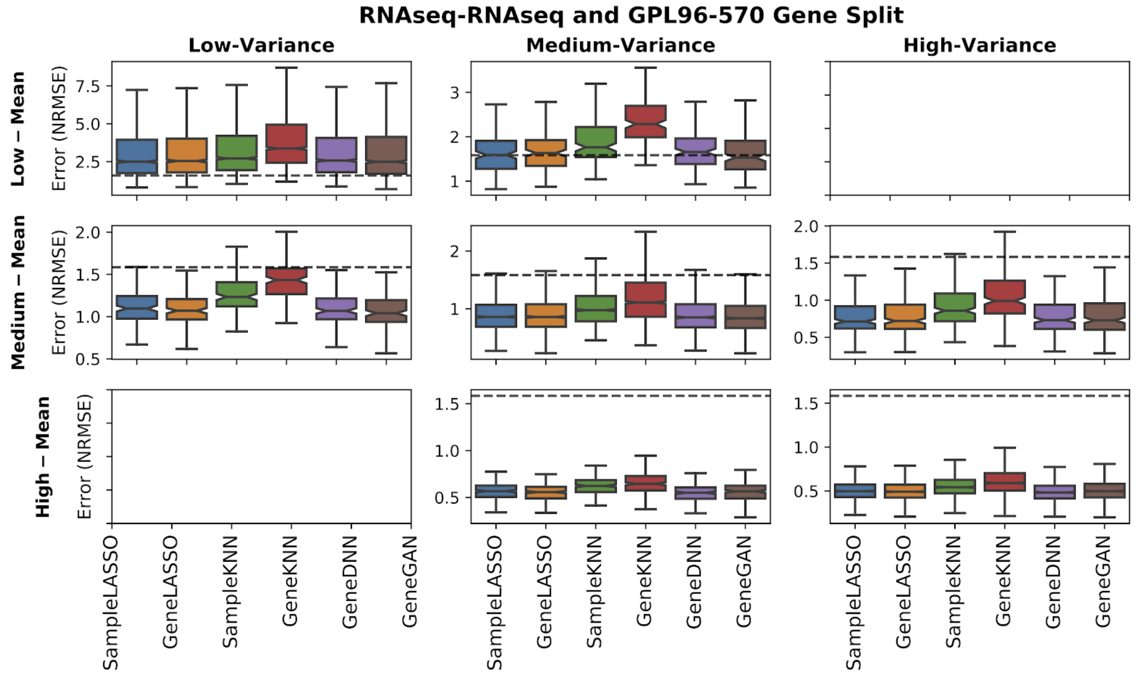

Fig. S24. Results broken up by mean and variance of gene expression for using RNA-seq data to impute RNA-seq data for the GPL96-570 gene subset. The dotted line is the median value when considering all genes for *SampleLASSO* (this is to help compare performances across the panels).

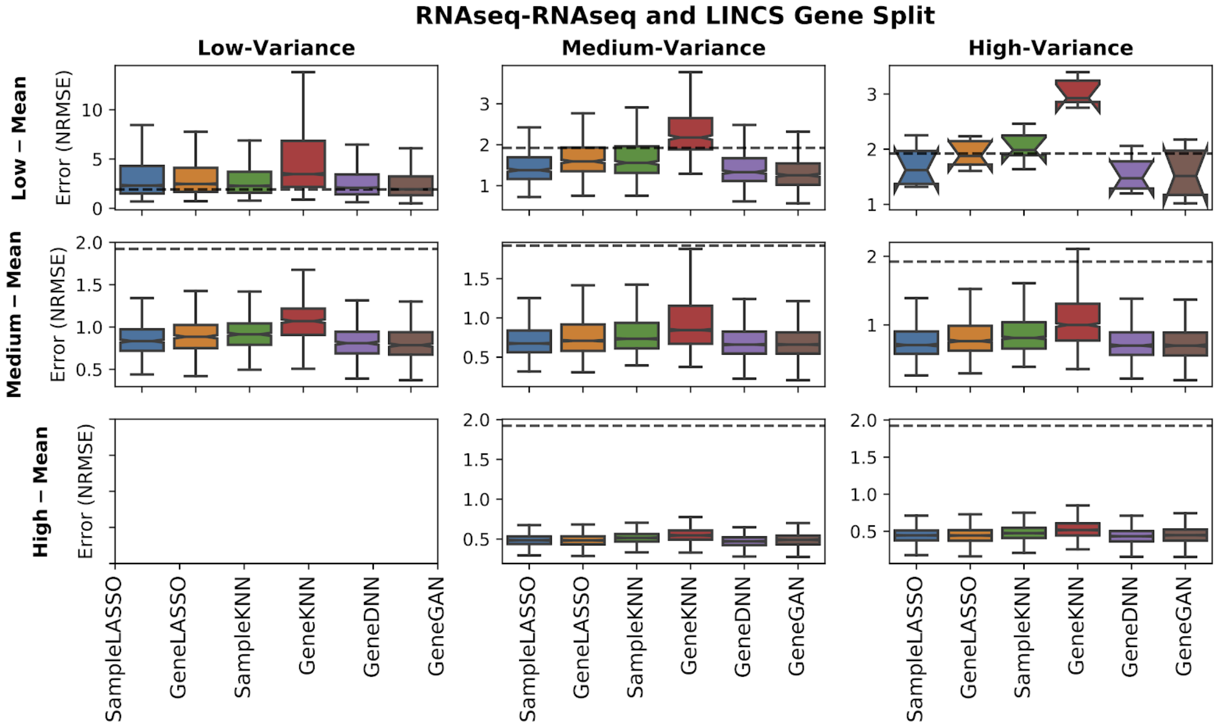

Fig. S25. Results broken up by mean and variance of gene expression for using RNA-seq data to impute RNA-seq data for the LINCS gene subset. The dotted line is the median value when considering all genes for *SampleLASSO* (this is to help compare performances across the panels).

Section 2.5: Supplemental Material for SEEK Analysis

To illustrate how much information is lost if we were to only consider the common genes when jointly analyzing data from two different platforms, we found the number of genes that would be thrown away when combining any two platforms in the SEEK database (i.e. the union minus the intersection of the genes in the two platforms). As can be seen in Figure S26, this usually results in losing information corresponding to thousands of genes.

As it was unfeasible to perform hyperparameter tuning for all methods for all ten platforms, we chose a single set of parameters to use for each method on all ten platforms. The hyperparameters were chosen by looking at all the results from the hyperparameter tuning analysis described in Section 1.4, and selecting the hyperparameter(s) that performed the best across all tasks and gene subsets for each method. This resulted in *k* being 10 for both *SampleKNN* and *GeneKNN*, alpha being 0.01 and 0.001 for *SampleLASSO* and *GeneLASSO*, respectively. For both deep learning methods the optimizer was set to Adam with a learning rate of 10^{-5} and 10^{-3} for *GeneDNN* and *GeneGAN*, respectively.

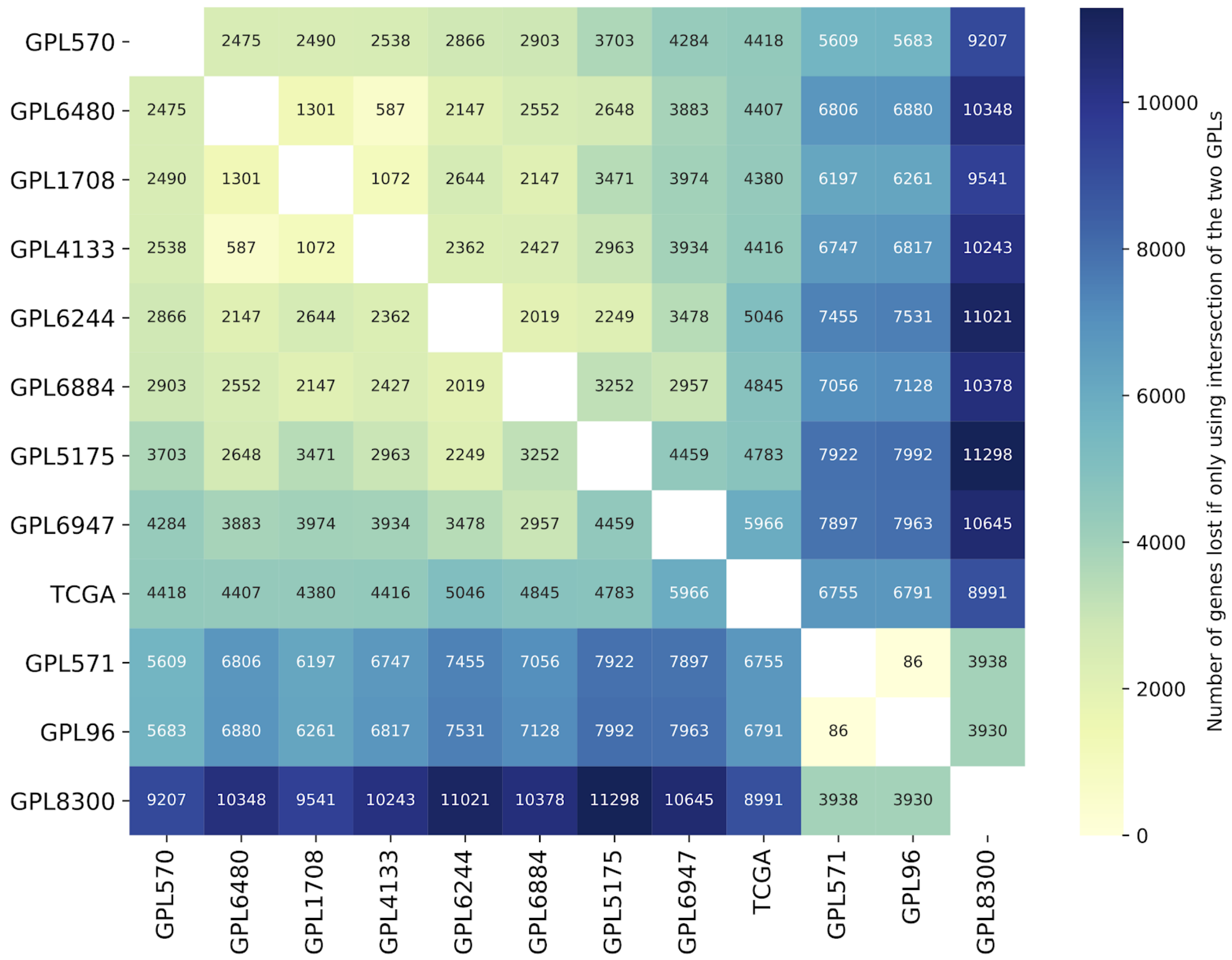

Fig. S26. Information lost when restricting analysis to genes common between two platforms. The values of the heatmap represent the union minus the intersection of the genes contained in a given pair of expression platforms from the SEEK database.

Section 2.6: Supplemental Material for Beta Analysis

The breakdown of the number of how many samples for each tissue were used in the beta-coefficient analysis can be found in Table S4. We additionally analyzed the beta-coefficients by grouping the z-scores of all non-target tissue samples together into a non-target group, as well as grouping the z-scores for all target tissue samples together. This was done for each tissue separately [Fig. S27]. An example of information returned by the Expresto software (https://github.com/krishnanlab/Expresto) can be seen in Table S5.

Table S4. Statistics of Data Used in Beta-Coefficient Analysis

	Test Set		Training Set	
**Tissue**	**Number of GSMs**	**Number of GSEs**	**Number of GSMs**	**Number of GSEs**
Blood	63	5	1197	33
Liver	40	6	776	27
Breast	40	6	770	25
Brain	39	3	752	19
Lung	29	6	662	10
Kidney	11	3	240	6

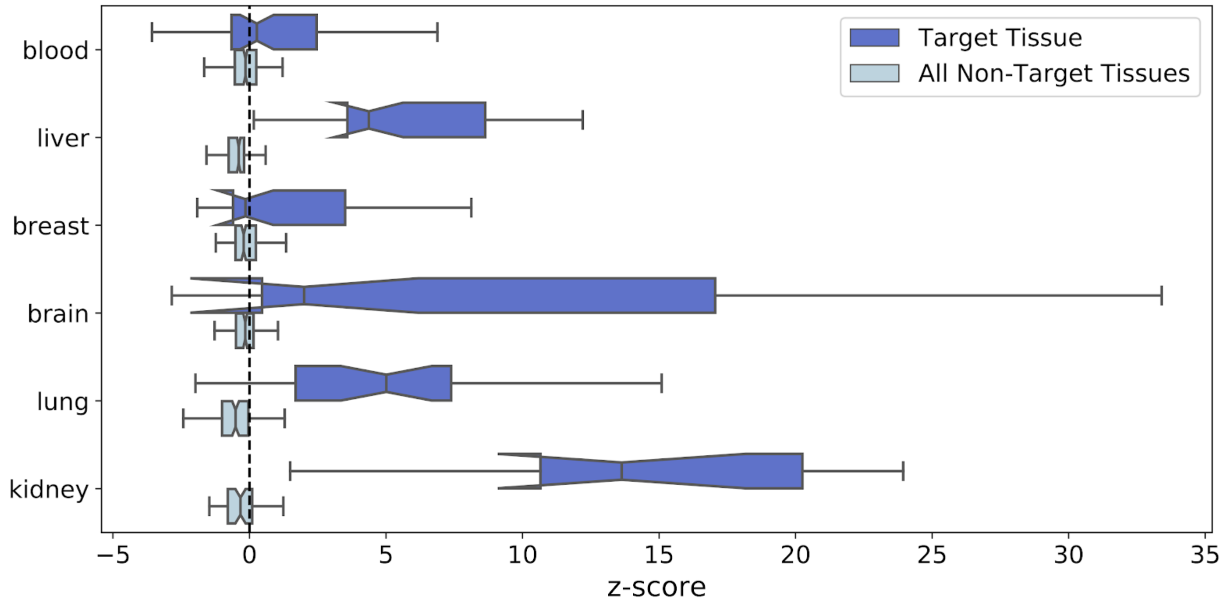

Fig. S27. Model interpretability of the target tissue versus rest of tissues combined. For each test sample labeled for a given tissue, we found the z-score for all six tissues considered. The boxplots show the distribution of z-scores for the target tissue, as well as all non-target tissues.

Table S5. Example of information that can be obtained from the user_function in the *Expresto* software released with this work

Target sample being imputed		Training samples with the three highest model coefficients			
			**First**	**Second**	**Third**
**Sample**	GSM478457	**Sample**	GSM175950	GSM388108	GSM388111
**Study**	GSE19279/GSE19281	**Study**	GSE7307	GSE15471	GSE15471
		**Beta-coeff**	0.34	0.15	0.14
**Annotation**	Title: Normal pancreas, 3 (U133A)	**Annotation**	Title: Pancreas SG1 Normal	Source name: pancreas	Source name: pancreas
**Sample**	GSM664048	**Sample**	GSM102499	GSM151315	GSM687049
**Study**	GSE26971	**Study**	GSE2109	GSE6532	GSE27830/GSE54219
		**Beta-coeff**	0.15	0.11	0.09
**Annotation**	Source name: primary breast cancer sample, fresh-frozen	**Annotation**	Title: Breast - 129692	Source name: breast	Title: primary breast cancer, sample_1927
**Sample**	GSM4005	**Sample**	GSM342677	GSM42736	GSM342884
**Study**	GSE475	**Study**	GSE13070	GSE2328	GSE13070
		**Beta-coeff**	0.15	0.13	0.12
**Annotation**	Source name: Human diaphragm	**Annotation**	Source name: skeletal muscle (vastus lateralis)	Source name: skeletal muscle	Source name: skeletal muscle (vastus lateralis)

Section 2.7: Loss Curves for *GeneDNN*

In this section, we present the loss curves for the *GeneDNN* [Fig. S28]. The data was generated using the *csv_logger* callback in *Keras* using the mean absolute error across all examples of the output of the mode. For all plots the training error displays the expected trends. Two interesting observations are for using RNA-seq data to impute RNA-seq data, the validation data has a lower loss than the training data. When using RNA-seq data to impute microarray data, the validation loss is much higher than the training loss, suggesting that the model is overfitting to the training data. We note that we do not show the loss curves for *GeneGAN* as, when we were writing the code for the *GeneGAN* method, we recorded these training loss curves incorrectly.

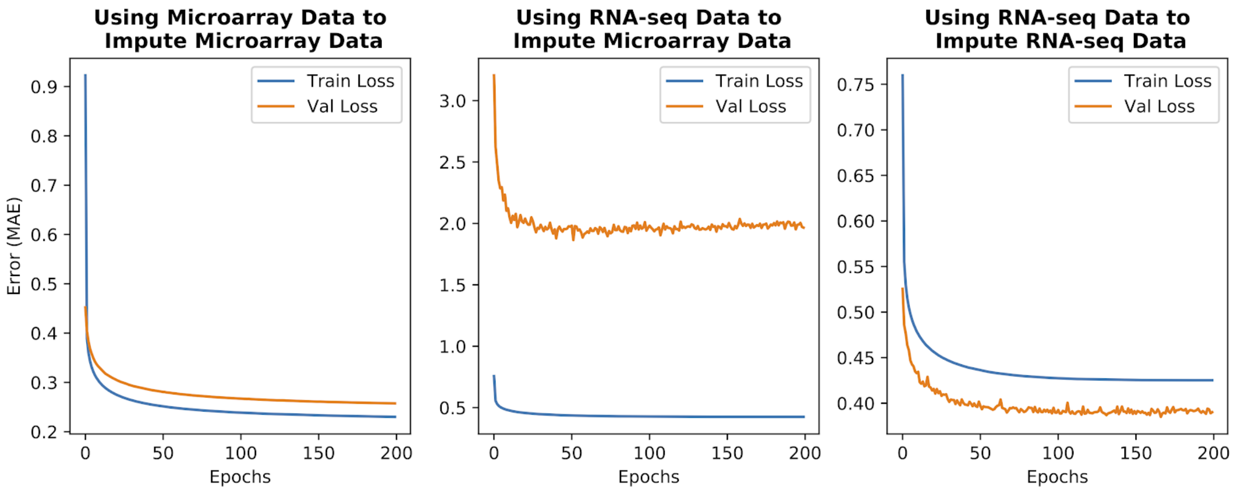

Fig S28. Loss curves the *GeneDNN* method on the LINCS gene subset.

References

1. McCall,M.N., Bolstad,B.M. and Irizarry,R.A. (2010) Frozen robust multiarray analysis (fRMA). *Biostatistics*, **11**, 242–253.
2. Dai,M., Wang,P., Boyd,A.D., Kostov,G., Athey,B., Jones,E.G., Bunney,W.E., Myers,R.M., Speed,T.P., Akil,H., *et al.* (2005) Evolving gene/transcript definitions significantly alter the interpretation of GeneChip data. *Nucleic Acids Res.*, **33**, e175–e175.
3. Chen,Y., Li,Y., Narayan,R., Subramanian,A. and Xie,X. (2016) Gene expression inference with deep learning. *Bioinformatics*, **32**, 1832–1839.
4. Wang,X., Ghasedi Dizaji,K. and Huang,H. (2018) Conditional generative adversarial network for gene expression inference. *Bioinformatics*, **34**, i603–i611.
5. Zhu,Q., Wong,A.K., Krishnan,A., Aure,M.R., Tadych,A., Zhang,R., Corney,D.C., Greene,C.S., Bongo,L.A., Kristensen,V.N., *et al.* (2015) Targeted exploration and analysis of large cross-platform human transcriptomic compendia. *Nat. Methods*, **12**, 211–214.
6. Glorot,X. and Bengio,Y. (2010) Understanding the difficulty of training deep feedforward neural networks. In *Proceedings of the Thirteenth International Conference on Artificial Intelligence and Statistics*.pp. 249–256.
7. Kingma,D.P. and Ba,J. (2017) Adam: A Method for Stochastic Optimization. *ArXiv14126980 Cs*.
8. Reddi,S.J., Kale,S. and Kumar,S. (2019) On the Convergence of Adam and Beyond. *ArXiv190409237 Cs Math Stat*.
9. Zeiler,M.D. (2012) ADADELTA: An Adaptive Learning Rate Method. *ArXiv12125701 Cs*.
10. Chollet,F. (2015) Keras.
11. Abadi,M., Agarwal,A., Barham,P., Brevdo,E., Chen,Z., Citro,C., Corrado,G.S., Davis,A., Dean,J., Devin,M., *et al.* (2016) TensorFlow: Large-Scale Machine Learning on Heterogeneous Distributed Systems. *ArXiv160304467 Cs*.
